# Reproductive senescence in a polymorphic raptor: phenotypic, sex, and environmental effects

**DOI:** 10.1093/beheco/arag024

**Published:** 2026-03-05

**Authors:** Elisa P Badás, Duarte S Viana, Jordi Figuerola, Laura Gangoso

**Affiliations:** Department of Evolutionary Ecology, National Museum of Natural Sciences—Spanish National Research Council (CSIC), C/José Gutiérrez Abascal 2, Madrid 28006, Spain; Department of Biodiversity, Ecology and Evolution, Faculty of Biological Sciences, Complutense University of Madrid, Antonio Novais 12, Madrid 28040, Spain; Estación Biológica de Doñana, CSIC, Américo Vespucio 26, Sevilla 41092, Spain; Estación Biológica de Doñana, CSIC, Américo Vespucio 26, Sevilla 41092, Spain; CIBER Epidemiología y Salud Pública (CIBERESP), Avenida Monforte de Lemos 3-5, Madrid 28029, Spain; Department of Biodiversity, Ecology and Evolution, Faculty of Biological Sciences, Complutense University of Madrid, Antonio Novais 12, Madrid 28040, Spain; Estación Biológica de Doñana, CSIC, Américo Vespucio 26, Sevilla 41092, Spain

**Keywords:** aging, developmental stress, Falconidae, fitness, fledgling success, high-quality environment, lifespan, raptor

## Abstract

The decline in reproductive performance with age (reproductive senescence) is a well-known process in evolutionary biology. Still, the interactive nature of factors operating at the individual level, such as sex or phenotype, and their effects on the rates of senescence, are understudied. Little is also known about the interaction between certain phenotypic traits and early-life conditions, despite the latter being known to impact fitness and senescence. Species that show genetically determined polymorphisms may aid to disentangle variation in senescence in response to environmental constraints, particularly when distinct physiological and behavioral responses are found across phenotypes. Here, using complete life histories gathered over 13 yr, we evaluated age, environmental and phenotypic effects on annual offspring number and lifetime reproductive success in a color polymorphic raptor, the Eleonora's falcon (*Falco eleonorae*). In females, we found evidence of within-individual reproductive senescence with differing patterns between color morphs, highlighting the need to further explore the sex-specific effects of overproducing melanin on senescence rates. We also found higher breeding success at older ages in both sexes, indicating population-level effects (selective appearance). Independent of sex and morph, higher lifetime reproductive success was associated with the exposure to a benign developmental environment, suggesting a “silver-spoon” effect of early-life conditions on fitness. However, in short-lived individuals, the negative effects of a harsh developmental environment on reproductive output were less evident. We propose that a breeding strategy that maximizes reproductive effort early in adulthood may be beneficial under unfavorable early-life conditions, at the expense of shorter lifespan.

## Teaser

Why individuals age differently in the wild is still puzzling, partly because the external and the genetic environment generate complex interactions. Using lifetime data from Eleonora's falcons, we found a decline in reproductive success with age in females that differed between the dark and the pale morphs. Moreover, falcons exposed to a benign environment early in life had more fledglings throughout their life. However, in the shorter-lived, a harsh environment early in life may not compromise reproduction.

## Introduction

Reproduction vs. physiological function tradeoffs are a central tenet in evolutionary ecology and life-history theory (Stearns 1992). In particular, the tradeoff between the increase in energy expenditure during reproduction to maximize the inheritance of genes vs. the depletion of resources available for self-maintenance is well understood and established across the animal kingdom (Healy et al. 2019). Individuals aim to adjust reproductive decisions to minimize the impact of reproduction on other life-history traits such as survival, longevity, or offspring size (Stearns 1992, 2000; Zera and Harshman 2001), and in doing so, they face internal and external challenges during their reproductive lifetime. For example, reproductive performance is known to profoundly affect senescence and vice versa across taxa, from microbes to vertebrates. With some exceptions (Jones et al. 2014), virtually all animals show a decline in reproductive function with age, a process known as reproductive senescence (Kirkwood and Rose 1991). In recent decades, multiple studies have shown evidence of age-related declines in Darwinian fitness in wild populations (for a review see Nussey et al. 2013), eg in birds (Bouwhuis et al. 2009), mammals (Dugdale et al. 2011; Hayward et al. 2015), and insects (Zajitschek et al. 2009). But despite such an increase in research effort to understand aging in the wild, the remarkable within-species differences in aging trajectories are still puzzling (Selman et al. 2012; Elliott et al. 2021; May 2023; Lemaître et al. 2024).

Phenotypic differences may explain the disparity in senescence trajectories across individuals within the same species. In this regard, genetically determined color polymorphism, whereby 2 or more distinct color morphs coexist in sympatric populations (Fisher 1930), may be particularly useful to study intraspecific variation in aging due to the strong between-morph variation in physiology and behavior. Morph-specific patterns are commonly reported in fitness (Brazill-Boast et al. 2013; Kappers et al. 2020), physiological parameters (Galván et al. 2010; Gangoso et al. 2011), immune response (Gangoso et al. 2015b), or molecular biomarkers of aging such as telomere length (Karell et al. 2017; Morosinotto et al. 2021). However, reproductive senescence studies in color-polymorphic species are lacking. A study in the dimorphic white-throated sparrow (*Zonotrichia albicollis*) reported morph-specific reproductive senescence in the white morph (Grunst et al. 2018a), but the authors did not distinguish within- from between-individual patterns and thus their results may be confounded by the selective disappearance of successful breeders at later ages (van de Pol and Wright 2009). To our knowledge, no study has assessed within-individual senescence trajectories across genetically determined color-morphs. Addressing this topic requires lifetime monitoring of individuals to capture morph-specific senescence patterns, yet such longitudinal datasets remain rare in natural populations.

Aside from coloration, a variety of intrinsic factors (eg sex) could interact to affect senescence trajectories. Evidence pointing toward sex-specific differences in aging is accumulating: males generally show shorter lifespan and an earlier onset/faster rate of actuarial senescence (ie the increase in mortality risk with age) in mammals, with the opposite pattern being found in birds (Grunst et al. 2018b, note sex- and morph-specific differences; Staerk et al. 2025). Differences in reproductive senescence patterns between sexes have, however, received less attention (see Lemaître and Gaillard (2023) and references therein), partly because the physiological/molecular mechanisms behind sex-specific aging are mostly unknown (Bronikowski et al. 2022). In fact, results are not always consistent across species: some studies did not find differences in reproductive senescence trajectories between males and females, while others found opposing patterns in different, but closely related taxa (see Comizzoli and Ottinger (2021) for a review).

The external environment could also shape senescence trajectories, because conditions are rarely ideal in wild populations. Even when decision-making is aimed at matching the optima via phenological adjustments (Schwartz 2003), extrinsic factors often interact in complex ways. Individuals are exposed to multiple stressors (eg limited resources, sibling competition, parent-offspring conflict, exposure to parasites/infectious diseases, environmental harshness) that ultimately affect fitness. Specifically, the early-life environment is known to be particularly challenging, and as such, it may have a long-lasting impact on performance (Gilbert 2001; Monaghan 2008). Nutritional stress experienced during development, eg negatively affects offspring survival across taxa (Buchanan et al. 2022), and it may drive adjustments in reproductive behavior (as suggested in long-lived seabirds [Kitaysky et al. 2010]) to adaptively avoid oxidative stress (Marasco et al. 2017) or the acceleration of aging-related processes, eg telomere shortening (Badás et al. 2015). But the interactive effects of the early-life environmental conditions on senescence are complex (Badás et al. 2023). In fact, the long-term effects of the interaction between certain phenotypic traits and early-life conditions on senescence rates remain unexplored when, eg color morphs have been found to cope differently with nutritional stress (in Eleonora's falcons *Falco eleonorae* the dark morph attains higher breeding success in unfavorable years; Gangoso and Figuerola 2019). Thus, major knowledge gaps in our understanding of the disparity in senescence trajectories include: (i) the impact of early-life vs. late-life environmental conditions on reproductive performance as individuals age, (ii) the effects of developmental vs. late-life stress on reproductive senescence, and (iii) the interaction between environmental effects and intraspecific phenotypic variation in shaping reproductive senescence patterns.

To address these gaps, we evaluated age, phenotypic, and environmental effects on reproductive senescence and lifetime reproductive performance using complete life-history data from a wild population of a polymorphic raptor, the Eleonora's falcon, breeding in the Canary Islands (Spain). The Eleonora's falcon is a long-distance migrant with the bulk of the breeding population distributed along the Eastern Mediterranean (Walter 1979). This species shows 2 discrete melanin-based color morphs (‘pale” and “dark’) under Mendelian inheritance, with the dark allele being dominant over the pale one (Gangoso et al. 2011). The aims of this study were 2-fold. First, according to the reproduction vs. self-maintenance tradeoff, we aimed at disentangling age and phenotypic (coloration) effects on reproductive senescence in both sexes. Second, we aimed to unravel the interactive effects of the early- vs. late-life environment on reproductive senescence trajectories. Previous studies have assessed how early-life conditions influence senescence patterns by testing 2 common hypotheses in evolutionary ecology: the “silver-spoon” hypothesis, which predicts that individuals raised under benign conditions outperform those from harsh environments (Cooper and Kruuk 2018); and the “predictive-adaptive response” hypothesis, which proposes that individuals whose adult-life environmental conditions match those experienced early in life should outperform those experiencing different early- vs. late-life conditions, irrespective of early-life environmental quality (Nettle et al. 2013). In addition to exploring these hypotheses, by specifically including the environment × phenotype × age interaction, we also test, for the first time (but see Spagopoulou et al. 2020 for a similar approach), whether phenotypic effects and/or the early- and the late-life environmental conditions shape differential reproductive senescence trajectories. In the population under study, dark morph nestlings show lower levels of the intracellular antioxidant glutathione (Galván et al. 2010) and mount weaker innate and acquired immune responses to some antigens (Gangoso et al. 2015b). Breeding and foraging strategies also appear to be morph-specific: dark males tend to be more territorial, achieve higher reproductive success than pale ones in some years (Gangoso et al. 2015a), and preferentially exploit alternative prey sources (Gangoso et al. 2024). Given the behavioral and physiological implications of the color phenotype, we predict morph-specific reproductive senescence patterns. In agreement with the “oxidative stress theory of ageing” (Finkel and Holbrook 2000), we expect the dark morph to show an earlier onset and a faster rate of reproductive senescence than the pale morph. Sex effects, on the other hand, are harder to predict due to the above-mentioned disparity in senescence rates found across species and sexes. Lastly, breeding success in this population is highly dependent on food availability controlled by trade winds, which largely determine the suitability of environmental conditions during the breeding season (Gangoso et al. 2020). Indeed, long and unexpected periods of nutritional stress (up to 12 d with no prey available) are common in the Canary Islands, exposing nestlings to quantifiable environmental stress during development (Gangoso et al. 2020). Thus, we predict the environment to shape reproductive senescence trajectories, possibly through a silver-spoon effect resulting from the long-lasting impact of developmental conditions on adult performance.

## Materials and Methods

### Study site and sampling

The study was conducted from 2007 to 2022 in a free-living population of Eleonora's falcon breeding in Alegranza (Chinijo Archipelago, Canary Islands, Spain, 29°24ʹ N, 13°30ʹ W, 10.2 km^2^, 289 m a.s.l.), which has been monitored yearly since 2007 (Gangoso et al. 2020). Every year, nest sites were routinely checked to assess egg laying, clutch size and fledgling number. Eleonora's falcons are highly philopatric birds (Ristow et al. 1980) that lay a single clutch per breeding season, ranging from 1 to 4 eggs (μ_2007–2022_ = 2.41, SD = 0.83, *N* = 886 in the Canarian population). Fledgling number ranges also from 1 to 4 (μ_2007–2022_ = 1.55, SD = 0.99, *N* = 886; note that in the study dataset, the maximum number of fledglings was 3). At the start of the season both members of the adult pair were identified by their uniquely numbered plastic rings using a telescope and their identity was confirmed using camera traps (Moultrie digital game camera, model M-40i) placed in the surroundings of the nest site during the incubation/early rearing period (when nestlings were ∼0 to 10 d old). Both members of the pair incubate the eggs. Males primarily assume provisioning duties to feed the female (during incubation) and the offspring, whereas females mainly focus on brood care (Gangoso et al. 2020). Near fledging (μ age in days = 29.19, SD = 5.19), nestlings were measured (wing length), weighed, and ringed with aluminum and plastic rings. Their exact age (±1 d) was calculated using the wing length according to the formula provided by (Ristow and Wink 2004) and laying date was calculated for each nest by subtracting 30 d (incubation period ±2 d) from the hatching date of the oldest nestling. Nestlings were color-ringed for the first time in the population in 2007. At the time of ringing, a few drops of blood were taken from the brachial vein using sterile syringes for molecular sexing following (Griffiths et al. 1996) and immediately stored in Eppendorf tubes filled with ethanol. Individuals are recruited into the population as breeders from 2 (in females) and 3 yr old (in males) onward and no differences are observed between morphs with regards to age of recruitment (Gangoso et al. 2013).

### Characterization of the early- and late-life environment

Environmental quality and/or environmental stress during breeding are often hard to quantify. In this population, though, the Atlantic trade wind patterns strongly determine food availability during the breeding season by regulating the flux of migrating passerines composing their diet, and consequently the population's breeding success (explaining up to 86% in mean annual productivity, Gangoso et al. 2020). We extracted wind data from the ERA5 fifth-generation reanalysis from the ECMWF—European Center for Medium-Range Weather Forecasts—for global climate and weather (Hersbach et al. 2020). Wind data (with directionality indicated by the west–east or *u* component, and the south–north or *v* component, m/s) were available hourly at a spatial resolution of 0.25° × 0.25° and were extracted for an area encompassing typical passerine migration paths between Southern Iberia and the Canary Islands (Gangoso et al. 2020) for the period 01 to 30 September each year from 2007 to 2022, the chick-rearing period of the Eleonora's falcon. We used the mean annual easterly wind intensity (u-wind component: μ = −0.70, SD = 1.51, range = [−3.23, 2.73]) as a proxy for the environmental quality that the falcons experience in the island. To characterize the early-life environment, we extracted the mean wind intensity during the breeding season of the year of birth, and to characterize the environment during adulthood (late-life environment) we extracted the mean wind intensity during the respective breeding season. Still, because wind intensity alone does not capture other minor aspects that may ultimately regulate productivity (total number of fledglings produced in the population for a given year divided by the number of breeding pairs monitored in the population), we also used the mean annual productivity, either of the year of birth (early-life environmental conditions) or of the breeding year (late-life environmental conditions) as a net measure of environmental quality. The analogous models using productivity as a proxy for the environmental quality are presented in Table S1 for the reproductive senescence models, and Table S2 for the lifetime reproductive success model).

### Statistical analyses

All analyses were performed in R version 4.2.3 (R Development Core Team 2023). First, we evaluated environmental and phenotypic effects on reproductive senescence and second, on lifetime reproductive performance:

#### Reproductive senescence models

To explore reproductive senescence trajectories in Eleonora's falcons we used annual offspring number (number of fledglings per breeding attempt) as dependent variable. In this population, given the environmental unpredictability during the breeding season (Gangoso et al. 2020), this metric is a better predictor of changes in reproductive success with age than other parameters such as clutch size, hatching success, or fledgling-to-egg ratios. Annual reproductive success rates are often under-dispersed (Brooks et al. 2019); therefore, we evaluated several error distributions (Poisson, Conway–Maxwell Poisson, generalized Poisson, negative binomial, and zero-inflated models) using the R package *glmmTMB* 1.1-7 (Brooks et al. 2017). Residuals were explored using the R package *DHARMa* 0.4-7 (Hartig 2024). Based on residual inspection, the best-performing models used a binomial error distribution, in which the response variable was coded as “successes vs. failures”. Using the R command “cbind” we input the successes as the number of fledglings per breeding attempt (0 to 3), and the failures corresponded to the remaining number of fledglings that the individual could have had in that breeding attempt but died at the nest (ie 3 in this dataset, minus the recorded number of fledglings). The models included the following fixed effects: age, quadratic age term (to capture senescence), morph, laying date, early-life environmental conditions (year of birth), and late-life environmental conditions (year of breeding attempt). All continuous variables were z-standardized (μ = 0, SD = 1). To distinguish between within-individual senescence and population-driven patterns (ie selective (dis)appearance from/into the population), and following (van de Pol and Wright 2009) we separated age effects. We included the “μ age” term (the mean age across all years sampled for a focal individual), which represents the between-individual differences, and the “δ age” term (the deviation between the focal individuals’ age at the year of breeding and the mean age), which represents the within-individual senescence trajectory. To test for phenotypic effects on reproductive senescence, we included the 2-way interactions between morph and each age term (δ or μ age); and to test whether the early- and late-life environmental conditions shape reproductive senescence trajectories, we included the 3-way interaction between the age term (δ or μ age) and early- and late-life environmental effects. Then, to select the most parsimonious model with regards to the fixed effects, we used the Akaike Information Criteria corrected for small sample sizes, AICc (Burnham et al. 2011) (Table S3). The random effects included: year of birth and year of breeding to control for temporal variation, colony (where the nest is located) to control for spatial variation, and individual identity to control for repeated measures on the same individuals. Marginal and conditional R^2^ values are presented for each model. In the results’ tables, parameter estimates are reported as the logarithmic values of the odds ratio, the usual output of logistic regression models. However, in the main text, for ease of interpretation, and because odds ratios do not directly correspond to probabilities (Davies et al. 1998), we report the marginal effects extracted using the R package *ggeffects* 2.2.0 (Lüdecke 2018), which are more biologically meaningful and comparable across studies. Full marginal effects are reported in Tables S4 to S7 as the back-transformed predicted probabilities of producing 3 fledglings per reproductive attempt. We built 2 reproductive senescence models, analyzed separately for female and male falcons because: (i) including sex as an independent effect in a single model would require complex 4-way interactions that are difficult to interpret, (ii) it would further reduce statistical power by increasing the sample size needed per estimated parameter, and (iii) it would complicate the random effects structure with a nested pair ID, risking model convergence.

Individuals with known birth year and at least 1 breeding attempt throughout their lifetime were included in the analyses (females: *N* = 103, *N*_breeding attempts_ = 256; males: *N* = 123, *N*_breeding attempts_ = 309). This meets the requirements proposed by van de Pol (2012), ie, a minimum sample size of *N* = 10 to 20 individuals and *N* ∼ 300 replicates to avoid bias and imprecision when estimating variance in slopes among individuals in random regression models (note that our aim was to estimate fixed effects, or mean slopes, rather than random effects, which would require larger datasets). In our dataset, the oldest female was 14 yr old (dark μ age = 5.38, SD = 2.70; pale μ age = 5.56, SD = 2.66) and the oldest male was 13 yr old (dark μ age = 5.53, SD = 2.09; pale μ age = 5.94, SD = 2.72); but see Table S8 for details on the structure of age classes. Reproductive performance data ranged from 1 to 8 breeding attempts in females (μ_females_ = 3.33, SD = 1.54) and up to 10 in males (μ_males_ = 3.20, SD = 1.64). Specifically, 65 of 103 females were monitored for more than 2 breeding attempts, and 24 for more than 4; for males, 83 of 123 were monitored for more than 2 attempts, and 24 for more than 4 (see Table S8 for details). However, because the sample sizes in this study were unbalanced between morphs (although balanced across age classes between morphs; Table S8), and to ensure that our results were not driven by data structure, unbalanced or low sample size, or influential points, we (i) used parametric bootstrapping (*n* = 1,000 samples with replacement) to obtain robust estimates, standard errors, and *P*-values; and (ii), we fitted cumulative ordinal mixed-effects models in a Bayesian framework using the R package *brms v*2.18.0 (Bürkner 2018), which yielded consistent results with the frequentist approach regarding the morph-dependent senescence trajectories (see Supplementary material for Bayesian methods and Tables S9, S10, and S11 for results). For simplicity, the main text presents only the frequentist approach with bootstrapped estimates, standard errors and *P*-values.

#### Lifetime reproductive success models

We evaluated the effects of early-life environment, lifespan, and phenotype on lifetime reproductive performance, using the total number of fledglings produced during an individual's lifetime (hereafter, LRS) as the dependent variable. Fixed effects included early-life environmental conditions, lifespan, morph, sex, and the interactions between early-life environment × lifespan × sex, and early-life environment × morph × sex. Continuous variables were z-standardized (μ = 0, SD = 1), and factors were mean-centered to facilitate interpretation of interaction terms. Year of birth was included as a random effect to account for temporal variation. A nest ID random term to control for partner effects on the breeding outcome was not included because about 70% of the adults in our dataset had 2 or more partners, and for the remaining 30%, the partner was not ringed (females, *N* = 106 pale, *N* = 12 dark; males, *N* = 112 pale, *N* = 29 dark). Competing models were fitted using *glmmTMB* as described for the reproductive senescence models, and the selected model used a negative binomial error distribution. The same model was run using mean annual productivity in the year of birth as a proxy for early-life environmental conditions (Table S2). To verify the model assumptions before interpretation, diagnostics plots were examined using the R package *DHARMa* 0.4-7 (Hartig 2024), and multicollinearity was assessed using variance inflation factors (VIF), all of which were <2. Robust parameter estimates are presented in the results tables, obtained following the same procedure described for the reproductive senescence models. To ensure the robustness of our conclusions from the LRS model, we also ran an analogous model using a dataset that only included individuals born before 2016 (given μ_observed lifespan_ = 6.32, SD = 2.89, ranging from 2 to 14 yrs), which gave identical results (Table S12).

## Results

### Age, phenotypic, and environmental effects on annual reproductive performance

**Figure 1:**
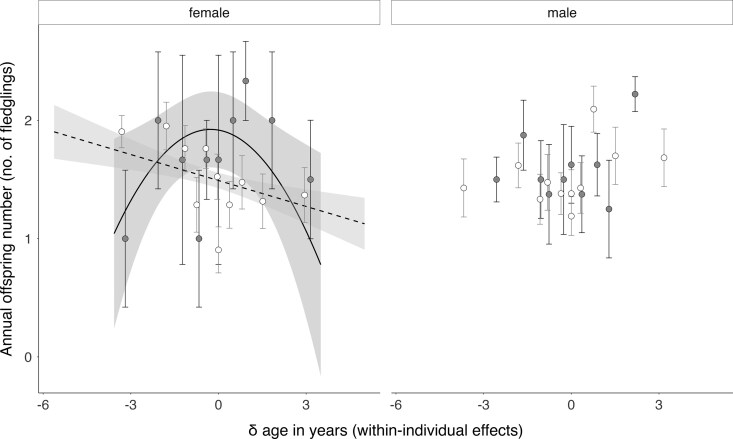
Within-individual (longitudinal) changes with age in annual offspring number (fledglings) in Eleonora's falcons. In females, lines represent the reproductive senescence pattern as the smoothed predicted values from the model with upper and lower 95% confidence intervals as shaded areas: solid line and dark gray shade for the dark morph; dashed line and light gray shade for the pale morph. No regression lines were plotted for males because no significant relationship was found. For visualization purposes, raw data points were binned and then averaged (thus, they do not reflect each within-individual's senescence pattern but mean slopes/patterns). Error bars represent standard errors, and points are plotted separately for the dark morph (gray circles with black error bars) and the pale morph (white circles with gray error bars).

In female Eleonora's falcons, the significant negative interaction between the quadratic δ age term and morph (Table 1) indicates a pattern of morph-dependent within-individual reproductive senescence: dark females show an initial increase followed by a later decline in reproductive performance with age (Fig. 1), a pattern that was not found in pale females, in which there was a linear decrease in reproductive performance with age (Fig. 1). The mean predicted peak in fledgling production for dark females occurred at age 5.36 (SD = 2.91), but note that this peak may vary among individuals despite the overall quadratic within-individual pattern. Thus, the marginal effects (lower CI, upper CI) predicted that dark females produced, on average, 1 (1, 2) fledgling 3 yrs before their reproductive peak; 2 (2, 3) fledglings at the peak, and 1 (1, 2) fledgling 3 yrs after the peak (full marginal effects for both morphs are shown in Table S4).

**Table 1:** Parameter estimates (bootstrapped) and random effects from the models explaining the effect of age, morph, and environmental conditions on annual reproductive performance of female and male Eleonora's falcons.

Predictors	Females				Males			
	β	SE	*Z*	*P*-value	β	SE	*Z*	*P*-value
(Intercept)	−1.61	0.30	−5.29	**<0.001**	−1.37	0.26	−5.28	**<0.001**
**Laying date**	**−0**.**22**	**0**.**09**	**−2**.**37**	**0**.**018**	−0.09	0.08	−1.18	0.238
Morph	−0.24	0.33	−0.73	0.468	−0.16	0.25	−0.62	0.536
δAge	0.01	0.06	0.19	0.848	0.09	0.05	1.75	**0**.**080**
δAge^2^	0.02	0.03	0.61	0.540	−0.03	0.03	−0.86	0.391
**μAge**	**0**.**48**	**0**.**12**	**3**.**99**	**<0.001**	**0**.**39**	**0**.**11**	**3**.**70**	**<0.001**
**μAge^2^**	**−0**.**03**	**0**.**01**	**−2**.**89**	**0**.**004**	**−0**.**02**	**0**.**01**	**−2**.**51**	**0**.**012**
**δAge^2^** **×** **Morph**	**−0**.**18**	**0**.**09**	**−2**.**02**	**0**.**044**	0.05	0.07	0.81	0.415
**μAge^2^** **×** **Morph**	**0**.**04**	**0**.**02**	**2**.**28**	**0**.**022**	0.00	0.01	−0.40	0.691
EL	0.02	0.10	0.18	0.854	−0.07	0.10	−0.66	0.507
**LL**	**−0**.**46**	**0**.**12**	**−3**.**85**	**<0.001**	**−0**.**57**	**0**.**11**	**−5**.**14**	**<0.001**
δAge^2^ × EL	−0.02	0.03	−0.78	0.435	0.08	0.05	1.70	**0**.**089**
δAge^2^ × LL	0.00	0.02	0.18	0.855	0.01	0.02	0.28	0.777
EL × LL	—	—	—	—	0.15	0.08	1.93	**0**.**054**
**Random effects**								
σ^2^	3.29				3.29			
τ_birth year_	0.00				0.00			
τ_year_	0.10				0.08			
τ_ID_	0.00				0.00			
τ_colony_	0.05				0.10			
*N*_birth year_	11				12			
*N*_year_	13				12			
*N*_ID_	103				123			
*N*_colony_	8				9			
Observations	256				309			
Marginal R^2^	0.182				0.164			

β, parameter estimate; SE, standard error; σ^2^, residual variance; τ, variance; CI, confidence interval; LL, late-life; EL, early-life. Laying date, EL environment and LL environment were standardized. The baseline level for morph was the pale morph. Note that the parameter estimates represent the logarithmic odds ratios of producing 3 fledglings per breeding attempt (in bold when significant). Significant predictors for either sex are highlighted in bold.

**Figure 2:**
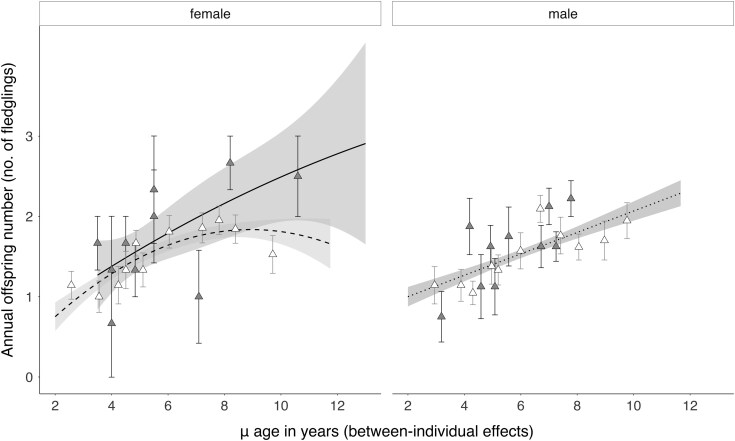
Between-individual (cross-sectional) changes with age in annual offspring number in Eleonora's falcons. Lines represent the smoothed predicted values from the models and upper with lower 95% confidence intervals shown as shaded areas: solid line and dark gray shade for dark morph females, dashed line and light gray shade areas for pale morph females. Note that for males only 1 regression line is plotted, because no differences were found across morphs. For visualization purposes, raw data points were binned and averaged, with error bars representing standard error: gray triangles and black error bars for the dark morph; white triangles and gray error bars for the pale morph.

Across females, we also found a morph-dependent senescence pattern, as indicated by the significant and positive effect of the μ^2^ age term in interaction with morph (Table 1, Fig. 2). This effect corresponds with the selective appearance of dark female breeders into the population at later ages: when the μ age was above 7 yr old (representing individuals sampled at more breeding events and older ages), dark females were predicted to produce 3 (2, 3) fledglings per reproductive attempt (marginal effects in Table S5). In other words, dark females that reached older ages were more successful breeders than pale females (Fig. 2). Conversely, successful pale female breeders selectively disappeared from the population (Fig. 2): at later ages pale females were predicted to produce 2 (1, 2) fledglings (μ age > 7 yrs, Table S5), or only 1 (0, 2) fledgling when even older (μ age > 10 yrs, Table S5). In addition to this, females were more likely to achieve higher annual reproductive success when they laid earlier in the season (Table 1, Figure S1). The model also confirmed that favorable adult-life environmental conditions were positively related with reproductive performance: on average, a 1 SD decrease in the mean annual wind intensity (more favorable winds; note that the *u*-wind component is negative) increased the likelihood of producing 3 fledglings by 20% (Table 1, Figure S2a and Table S6 for the marginal effects).

In male falcons, the strong between-individual age effect (Table 1, μ age β estimate = 0.39, Fig. 2) may have masked any within-individual reproductive senescence pattern (Table 1, δ age β estimate = 0.09; but note that, independent of morph, there was a trend toward a within-individual reproductive senescence pattern in interaction with the early-life environmental conditions, Figure S3). The quadratic pattern in the μ age term was also significant, but this effect was weak (Table 1, μ^2^ age β estimate = −0.02, Fig. 2), indicating that it could be driven by the initial increase in reproductive performance with increasing age in the population. Irrespective of morph, older males were predicted to produce 2 (1, 2) fledglings per attempt at a μ age above 7 yrs (Table S5). In other words, at the population-level, there was a strong pattern of selective appearance of successful male breeders at later ages (Fig. 2). In addition to this, as in females, favorable environmental conditions in the breeding year were a strong predictor of high annual reproductive success in males (Table 1, Figure S2b, and Table S6 for the marginal effects). There was also a trend suggesting that annual breeding performance was shaped by the interaction between early- and adult-life environmental conditions (Table 1, Figure S4).

### Lifespan, phenotypic and environmental effects on lifetime reproductive success (LRS)

**Figure 3:**
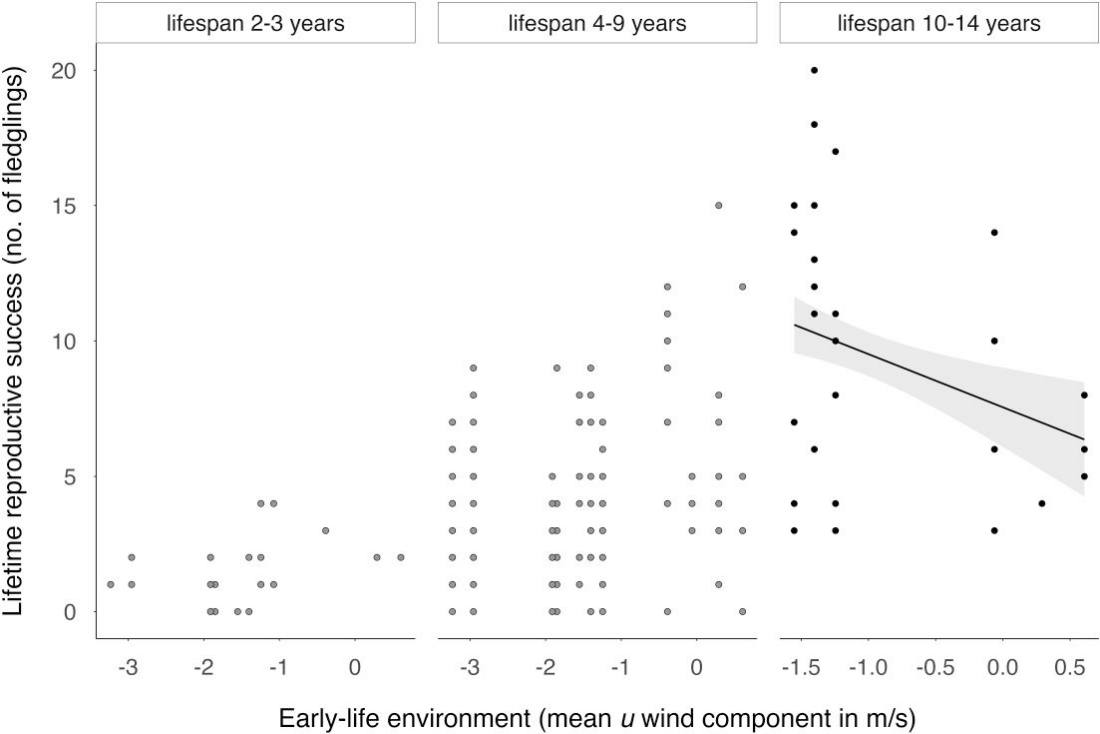
Lifetime reproductive success (LRS) in relation to lifespan and early-life environment for Eleonora's falcons. For graphical purposes, lifespan (in years) was categorized into 3 groups using the 25th percentile, the median, and the 75th percentile. Regression lines from the smoothed predicted values from the mixed model, 95% confidence intervals (shaded area), and raw data points are shown. Only the smoothed regression line for the significant group is plotted (early-life environment × lifespan 10 to 14 yrs: β = −0.52, SE = 0.24, Z = −2.18, *P*-value = 0.0292). Note that negative values of the mean west–east winds (*u* component) reflect favorable winds for the Eleonora's falcon and correlate with higher productivity (see Methods section).

**Figure 4:**
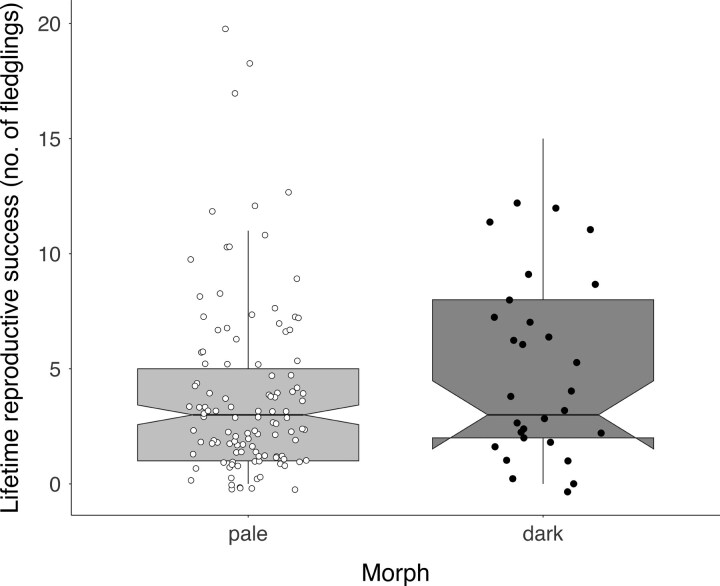
Lifetime reproductive success (LRS) in relation to morph in male Eleonora's falcons. Horizontal lines in boxplots represent the median, upper and lower quartiles and whiskers represent the 1.5× the interquartile range. Raw data points (jittered to prevent overlap) are shown (in black for the dark morph and white for the pale morph).

On average, LRS was strongly and positively related to lifespan (Table 2), with marginal effects predicting 1 [1, 2], 3 [3, 4], and 7 [7, 10] fledglings for individuals with 2, 5 and 9 yrs of observed lifespan, respectively. Early-life environmental conditions were also associated with LRS, but this relationship was lifespan-dependent, as indicated by the significant interaction between lifespan and early-life conditions (Table 2, Fig. 3). Independent of sex, in longer-lived individuals, higher LRS correlated with favorable early-life conditions: eg 9 [7, 14] fledglings were predicted when the *u*-wind component was −3.23 m/s, while 6 [5, 10] fledglings were predicted when the u-wind component was 0.61 m/s (Table S7 for full marginal effects). However, in shorter-lived individuals this relationship was absent (Fig. 3). In addition, dark morph falcons achieved marginally significantly higher LRS (marginal effects = 5 [4, 7] fledglings) than pale ones (marginal effects = 4 [4, 5] fledglings, Table 2, Fig. 4).

**Table 2:** Parameter estimates (bootstrapped) and random effects from the generalized mixed model explaining the effect of lifespan, morph, and early-life environmental conditions on lifetime reproductive performance of female and male Eleonora's falcons.

Predictors	β	SE	z	*P*-value
(Intercept)	1.22	0.07	17.30	**<0.001**
EL environment	0.07	0.06	1.19	0.234
**Morph**	**0**.**22**	**0**.**11**	**2**.**00**	**0**.**046**
Sex	−0.04	0.09	−0.49	0.624
**Observed lifespan**	**0**.**67**	**0**.**05**	**12**.**78**	**<0.001**
EL environment × morph	0.06	0.12	0.48	0.631
EL environment × sex	−0.01	0.09	−0.16	0.874
Morph × sex	0.04	0.21	0.17	0.863
**EL environment × observed lifespan**	**−0**.**16**	**0**.**06**	**−2**.**47**	**0**.**014**
Sex × observed lifespan	0.09	0.09	1.01	0.311
EL environment × morph × sex	0.10	0.23	0.44	0.658
EL environment × sex × observed lifespan	0.05	0.12	0.42	0.673
**Random effects**				
σ^2^	0.27			
τ_birth year_	0.02			
ICC	0.08			
*N*_birth year_	12			
Observations	259			
Marginal R^2^/conditional R^2^	0.593/0.626			

β, parameter estimate; SE, standard error; σ^2^, residual variance; τ, variance; ICC, interclass correlation coefficient; EL,early-life. The baseline level for morph was the pale morph. Morph and sex were mean centered and EL environment was standardized. Note that parameter estimates represent the logarithmic odds ratios of producing an increasing number of fledglings throughout an individual's lifetime. Significant predictors are highlighted in bold.

## Discussion

This study provides the first evidence in a color-polymorphic raptor that within-individual reproductive senescence varies according to sex and morph, and that LRS may be shaped by environmental conditions experienced early in life. Previous research on color-polymorphic species has reported morph-specific differences in annual breeding performance and LRS (Krüger and Lindström 2001; Brommer et al. 2005; Kappers et al. 2020; Morosinotto et al. 2020; Tooth et al. 2024), yet most studies have not disentangled between- from within-individual effects, leading to conclusions on senescence that may be confounded by population-level patterns (van de Pol 2012). By analyzing complete life histories over 13 yr, we show that female Eleonora's falcons exhibit age-related declines in reproductive performance with morph-dependent trajectories: dark females display an initial increase in annual reproductive success followed by a decline, while pale females show a more linear decrease. In contrast, senescence patterns in males were less evident, and population-level processes, such as the selective appearance of successful breeders played a stronger role. These results highlight the importance of considering both intrinsic traits and extrinsic constraints when evaluating aging in wild populations.

The observed sex differences in within-individual reproductive senescence may be related to different roles during breeding. In this species, males undertake most of the food provisioning during the chick-rearing period (Gangoso et al. 2020). In raptors, younger individuals generally show low hunting success, a skill that improves markedly with age (Newton 1979; Rutz et al. 2005 and references therein). Consequently, hunting experience could play a key role in the selective appearance of successful male breeders at older ages, potentially obscuring within-individual senescence in this sex. Sex-specific senescence patterns have been reported across vertebrates (for a review, see Bronikowski et al. 2022), but the mechanisms underlying these differences remain largely unknown. Although speculative, an alternative (and not mutually exclusive) explanation why the senescence pattern was only found in females, and was more pronounced in the dark morph (quadratic vs. linear decline in the pale morph), could be an additive interactive effect between sex-specific hormones and melanin production. On the one hand, glucocorticoid hormones are key regulators of life-history strategies, and their levels have been shown to differ between sexes (O’Reilly and Wingfield 2001) and melanin-based color morphs (Roulin et al. 2008; Pryke et al. 2012). On the other hand, glutathione, a key endogenous antioxidant, is involved in melanin synthesis (Galván and Alonso-Alvarez 2009). In Eleonora's falcons, the genetically determined overproduction of eumelanin in the dark morph (Gangoso et al. 2011) may thus entail physiological costs (eg an increased intake of exogenous antioxidants or synthesis of endogenous antioxidants), as suggested for the booted eagle *Hieraaetus pennatus* (Galván et al. 2010). Indeed, dark morph nestlings in the study population have significantly lower levels of circulating glutathione (Galván et al. 2010). However, these hypotheses remain to be tested, eg by longitudinally measuring glucocorticoid levels and antioxidant markers across age classes, sexes, and morphs (Morosinotto et al. 2025).

Interestingly, taken together, our current and previous studies in this same population suggest that the 2 distinct breeding strategies observed between morphs may be linked to the reported morph-specific differences in reproductive senescence. Dark morph individuals occupy dominant social positions but display less aggressive behavior than their pale conspecifics (Gangoso et al. 2015b), and they achieve higher LRS (this study, Gangoso et al. 2015a; Gangoso and Figuerola 2019). Several mechanisms could explain this fitness advantage: the dark morph may compensate for low-quality years with (i) greater flexibility, eg by shifting to alternative prey such as local seabirds (in this population, dark morph males are more likely to hunt petrels (Gangoso et al. 2024), or (ii) by increasing parental care (Morosinotto et al. 2025). This agrees with our result that, at the population level, successful dark females selectively appeared into the breeding population at older ages. As opposed to this, the pale morph attains lower LRS, breeds colonially and is more aggressive (Gangoso et al. 2015a). Higher density of conspecifics increases the risk of intraspecific predation of neighboring chicks (Gangoso et al. 2015a), which may in turn increase maternal care costs for the females breeding in such colonies, who care for the offspring (Gangoso et al. 2020). This could explain our result that successful pale females, at the population-level, selectively disappeared from the breeding population. Similar phenomena of selective disappearance of high-quality individuals have been reported across a variety of species in wild populations, particularly birds such as barn swallows *Hirundo rustica* (Balbontin et al. 2007), mute swans *Cygnus olor* (McCleery et al. 2008), and great tits *Parus major* (Bouwhuis et al. 2009) as well as mammals such as the European badger *Meles meles* (Dugdale et al. 2011). Here, we were able to detect both within-individual and population-level effects that can be explained by morph-specific breeding strategies.

Our study did not find an *independent* positive relationship between the quality of the early-life environment and LRS, despite “silver-spoon” effects being widespread across taxa. For example, a meta-analysis that included 14 long-lived bird and mammal species reported a strong “silver-spoon” effect on reproductive senescence (Cooper and Kruuk 2018). Instead, in Eleonora's falcons, the association between developmental conditions and LRS was *dependent* on lifespan, because it was only found in long-lived falcons. Being exposed to a harsh environment early in life compromised LRS in long-lived falcons but not in the shorter-lived, indicating that some individuals may be able to adopt an adaptive breeding strategy to compensate the unfavorable developmental conditions. An adaptive shift in reproductive behavior (eg advancement) triggered by low-quality early-life conditions has been proposed by the future life expectancy hypothesis (Nettle et al. 2013, 2015) and reported in another raptor species, the Mauritius kestrel *Falco punctatus* (Cartwright et al. 2014); but it may be costly to maintain. Instead of advancing the age of first reproduction, we suggest that some Eleonora's falcons may adaptively increase reproductive effort to compensate an unfavorable upbringing, and this strategy provides a fitness benefit at the expense of a shorter lifespan. To our knowledge, this is the first time that a study reports a lifespan-dependent relationship between early-life conditions and late-life reproductive success.

Finally, the late-life environmental conditions were strongly associated with annual reproductive success in Eleonora's falcons (in this and previous studies in the population—Gangoso et al. 2020, 2024). Despite this, and the above-mentioned effects of the early-life environment on LRS, we did not detect an interactive effect between early- and late-life environmental conditions in annual reproductive success (but note the trend found in males), which would be in accordance with the predictive-adaptive hypothesis (Monaghan 2008; Nettle and Bateson 2015). As expected, unpredictable and/or highly variable environmental conditions during adulthood could preclude the predictive potential in long-lived species (Monaghan 2008). This could be the case in the Eleonora's falcon population breeding in the Canary Islands, where extreme climatic events are common: unfavorable winds may result in long-lasting food gaps (Gangoso et al. 2020). It should also be taken into account that some adult pairs might breed in inaccessible sites within the island, despite the high philopatry and site-tenacity reported for the species (Ristow et al. 1980). Such limitation could have obscured within-individual senescence patterns in males by reducing the number of monitored breeding occasions and limiting statistical power to detect stronger effects. Future studies should explore the differential survival probabilities of individuals with different breeding capabilities, to further confirm whether population-level effects mask stronger within-individual senescence effects (McCleery et al. 2008; Bouwhuis et al. 2009). In addition, further studies are needed on the physiological costs of harsh early-life conditions in combination with measures/manipulation of melanin/glucocorticoids, because these will shed light on within-individual senescence rates in polymorphic species. Nonetheless, our results have highlighted (i) the need to partition age into within- and between-individual effects, and (ii), the need to incorporate the relevant interactive effects between early-life environmental conditions and other life-history traits (eg lifespan). Without these, reproductive senescence studies could fail to detect adaptive breeding strategies that may arise to counteract the long-lasting effects of unfavorable developmental conditions.

## Supplementary Material

arag024_Supplementary_Data

## Data Availability

Data from this manuscript are available at https://doi.org/10.5281/zenodo.18376997. Analyses reported in this article can be reproduced using the data provided by Badás (2026). Statistical R code for this manuscript is available from Github (https://github.com/elisa-P-badas).
