# Supplementary material for: Reproductive senescence in a polymorphic raptor: phenotypic, sex, and environmental effects
Source: Behav Ecol. 2026 Mar 5;37(3):arag024. doi: 10.1093/beheco/arag024 (PMC13016796; doi:10.1093/beheco/arag024)

**Supporting Information for:**

**A polymorphic raptor reproducing against all odds: phenotypic,**

**sex and environmental effects on senescence**

Elisa P. Badás, Duarte S. Viana, Jordi Figuerola & Laura Gangoso

**Table of Contents:**

| **Supplementary information**  **for the statistical methods** | Pages 2-3 |
| --- | --- |
| **Supplementary tables** | Pages 4 - 10 |
| **Supplementary figures** | Pages 11 - 14 |

**Supplementary information for the statistical methods**

**Bayesian approach for the annual offspring model**

To distinguish between within-individual (i.e. senescence) and population-driven patterns (i.e. selective (dis)appearance from/in the population), we separated age effects (van de Pol & Wright, 2009) by including ‘average age’ (the mean age across all years sampled for a given individual, representing between-individual differences) and ‘delta age’ terms (the deviation of each individuals’ age at the year of breeding from the mean age across all years sampled for a given individual, representing within-individual senescence). In accordance with the two-fold study aims, to test phenotypic effects on reproductive senescence we included the 2-way interactions between morph and each separated age term (delta or average age); and to test whether the interplay between the early- and late-life environmental conditions and/or the phenotype shape reproductive senescence trajectories, we included the 3-way interactions between: (i) the age term and early- and late-life environmental effects and (ii), the colour morph and early- and late-life environmental effects. Thus, we used a model selection approach (see Table S1 for model specifications). Briefly, following van de Pol and Wright (van de Pol & Wright, 2009), model 1 included the linear and quadratic age terms, the 2-way interaction between the age terms and morph and annual productivity of the breeding year. Model 2 included linear and quadratic age-effects, but these were separated in delta and average age, their 2-way interactions with morph and the 2-way interaction between early and late-life environmental conditions. Model 3 retained the 2-way interaction between the quadratic age term and morph and removed the 2-way interaction between the linear age term and morph. Subsequently, model 4 included the same variables as in model 3 with the addition of the 3-way interaction between the quadratic delta age term, early-life and late-life environmental conditions; and model 5 included the same variables as in model 3 with the addition of the 3-way interaction between the quadratic average age term, early-life and late-life environmental conditions. Models 4 and 5 included the 3-way interaction between the quadratic (delta and average) age terms and the environmental conditions separately because the full model rendered over-parameterization issues (divergent transitions after warm up and sampling problems were detected in the diagnostic plots). Models 6 and 7 included the same predictors as models 4 and 5 except that we removed the 2-way interaction between the separated age effects and morph. All models included the following random effects: year of breeding to control for temporal variation, colony (where the nest is located) to control for spatial variation, and individual identity nested in year of birth to control for repeated measures on the same individuals. Subsequently, competing models were compared using leave-one-out cross-validation with the *loo* package v2.6.0 (Vehtari *et al.*, 2017) and the best model was selected when the expected Log pointwise predictive density (ELPD) difference and the standard error (SE) difference between an increasingly complex model and the previous one was <2 (Sivula *et al.*, 2020), and competing models are shown in Table S3. The model in which the early and late-life environments were characterised using mean annual productivity is shown in Table S4. The model including mean annual wind intensity gave similar results (Table S5). The Bayesian R^2^ is presented for each model. If the ELPD and SE differences were higher, the model with fewer parameters was selected. For population- level (fixed) effects, we used ‘weakly informative’ priors (mean = 0, SD = 1). For group- level (random) effects, we used the default priors of *brms* (half-t density with 3 degrees of freedom) for standard deviations. For each model, we ran four chains with 1,000 discarded warm-up iterations, followed by 5,000 sampling iterations, thus yielding 20,000 posterior samples per model. Proper mixing of chains was monitored with trace plots and convergence of chains by verifying that Ȓ values were close to 1.00. In all competing models, bulk and tail estimates of effective sample sizes were >5,000 for each predictor. Posterior predictive checks using the pp_check() function of the *brms* package confirmed that model fits performed well.

**Table S1.** Parameter estimates and random effects from the models explaining age, morph and environmental effects on annual reproductive performance of female and male Eleonora’s falcons. β = parameter estimate, SE = standard error, σ^2^ = residual variance, τ = variance, ICC = interclass correlation coefficient, CI = confidence interval, LL = late-life, EL = early-life. Laying date, EL environment and LL environment were standardised. Environmental conditions were characterised using the mean annual productivity. The baseline level for morph was the pale morph.

|  | **Females** | | | | **Males** | | | |
| --- | --- | --- | --- | --- | --- | --- | --- | --- |
| *Predictors* | β | *SE* | *Z* | *P-value* | β | *SE* | *Z* | *P-value* |
| (Intercept) | -1.70 | 0.60 | -2.85 | **0.004** | -1.61 | 0.74 | -2.19 | **0.029** |
| **Laying date** | **-0.22** | **0.09** | **-2.39** | **0.017** | -0.10 | 0.07 | -1.34 | 0.181 |
| Morph | -0.31 | 0.49 | -0.64 | 0.524 | -0.23 | 0.38 | -0.62 | 0.533 |
| δAge | 0.03 | 0.06 | 0.49 | 0.622 | **0.10** | **0.05** | **2.18** | **0.030** |
| δAge^2^ | 0.00 | 0.02 | 0.24 | 0.812 | 0.01 | 0.02 | 0.40 | 0.690 |
| **μAge** | **0.52** | **0.20** | **2.59** | **0.010** | 0.44 | 0.23 | 1.87 | **0.062** |
| **μAge^2^** | **-0.03** | **0.02** | **-2.20** | **0.028** | -0.03 | 0.02 | -1.46 | 0.144 |
| δAge^2^ × Morph | -0.14 | 0.08 | -1.81 | **0.070** | 0.03 | 0.06 | 0.45 | 0.653 |
| **μAge^2^ × Morph** | **0.03** | **0.02** | **2.01** | **0.044** | 0.00 | 0.01 | 0.10 | 0.917 |
| EL | -0.08 | 0.30 | -0.27 | 0.786 | -0.34 | 0.29 | -1.14 | 0.253 |
| **LL** | **1.46** | **0.24** | **6.05** | **<0.001** | **1.49** | **0.24** | **6.30** | **<0.001** |
| δAge^2^ × EL | 0.06 | 0.06 | 0.95 | 0.345 | 0.04 | 0.06 | 0.75 | 0.454 |
| δAge^2^ × LL | 0.00 | 0.05 | 0.03 | 0.975 | 0.02 | 0.05 | 0.48 | 0.630 |
| **Random Effects** | | | | | | | | |
| σ^2^ | 3.29 | | | | 3.29 | | | |
| τ _birth year_ | 0.00 | | | | 0.00 | | | |
| τ _year_ | 0.00 | | | | 0.01 | | | |
| τ _ID_ | 0.00 | | | | 0.00 | | | |
| τ _colony_ | 0.04 | | | | 0.107 | | | |
| N _birth year_ | 11 | | | | 12 | | | |
| N _year_ | 13 | | | | 12 | | | |
| N _ID_ | 103 | | | | 123 | | | |
| N _colony_ | 8 | | | | 9 | | | |
| Observations | 256 | | | | 309 | | | |
| Marginal R^2^ | 0.194 | | | | 0.178 | | | |

**Table S2.** Parameter estimates (bootstrapped) from the alternative generalised mixed models explaining lifespan, morph and environmental effects on lifetime reproductive performance of female and male Eleonora’s falcons. Here, environmental effects were characterised using the mean annual productivity. β = parameter estimate, SE = standard error, σ^2^ = residual variance, τ = variance, ICC = interclass correlation coefficient, EL = early-life. Note that parameter estimates represent the logarithmic odds ratio of producing an increasing number of fledglings throughout an individual’s lifetime. Significant parameter estimates are highlighted in bold.

|  |  | | | |
| --- | --- | --- | --- | --- |
| *Predictors* | β | *SE* | *Z* | *P - value* |
| (Intercept) | 1.17 | 0.06 | 18.24 | **<0.001** |
| EL environment | -0.11 | 0.06 | -1.84 | 0.066 |
| **Morph** | **0.22** | **0.11** | **2.04** | **0.042** |
| Sex | 0.00 | 0.08 | -0.06 | 0.956 |
| **Observed lifespan** | **0.61** | **0.04** | **14.70** | **<0.001** |
| EL environment × Morph | 0.01 | 0.10 | 0.09 | 0.926 |
| EL environment × Sex | 0.00 | 0.08 | 0.04 | 0.964 |
| Morph × Sex | 0.06 | 0.19 | 0.31 | 0.757 |
| **EL environment × Observed lifespan** | **0.13** | **0.04** | **3.03** | **0.002** |
| Sex × Observed lifespan | 0.13 | 0.07 | 1.78 | 0.076 |
| EL environment × Morph × Sex | -0.08 | 0.18 | -0.41 | 0.684 |
| EL environment × Sex × Observed lifespan | 0.06 | 0.07 | 0.77 | 0.444 |
| **Random Effects** | | | | |
| σ^2^ | 0.27 | | | |
| τ _birth year_ | 0.02 | | | |
| ICC | 0.07 | | | |
| N _birth year_ | 12 | | | |
| Observations | 259 | | | |
| Marginal R^2^ / Conditional R^2^ | 0.587 / 0.618 | | | |

**Table S3:** Fixed effects that were included in the glmmTMB model selection to evaluate early- and late-life environmental, age and morph effects on annual reproductive performance of male and female Eleonora’s falcons. Key: LL = late-life environmental conditions (productivity of breeding year); EL = early-life environmental conditions (productivity of year of birth); δAge = delta age, representing within-individual age effects; μAge = average age, representing between-individual age effects; ELPD diff = Expected Log pointwise Predictive Density difference; SE diff = standard error difference. Selected models for each sex are highlighted in bold.

|  |  |  |  |  | | |  |  |  | |  |  |  |
| --- | --- | --- | --- | --- | --- | --- | --- | --- | --- | --- | --- | --- | --- |
|  |  | **Females** | | | | | | **Males** | | | | |  |
|  |  | Productivity  models | | | Wind  models | | | Productivity  models | | Wind  models | | |  |
| *Model no.* | *Predictors* | *AICc* | *weight* | | *AICc* | *weight* | | *AICc* | *weight* | | *AICc* | *weight* |  |
| 1 | δAge + δAge^2^*Morph + μAge + μAge^2^*Morph + Laying date + μAge^2^*EL*LL | 662.71 | 0.009 | | 702.83 | 0.000 | | 795.23 | 0.004 | | 827.77 | 0.000 |  |
| 2 | δAge + δAge^2^*Morph + μAge + μAge^2^*Morph + Laying date + δAge^2^*EL*LL | 659.43 | 0.046 | | 694.22 | 0.001 | | 790.28 | 0.042 | | 815.95 | 0.017 |  |
| **3** | **δAge + δAge^2^*Morph + μAge + μAge^2^*Morph + Laying date + δAge^2^*EL + δAge^2^*LL + EL*LL** | 655.57 | 0.318 | | 686.19 | 0.054 | | 786.31 | 0.304 | | **808.88** | **0.591** |  |
| 4 | δAge + δAge^2^*Morph + μAge + μAge^2^*Morph + Laying date + μAge^2^*EL + μAge^2^*LL + EL*LL | 657.38 | 0.128 | | 691.08 | 0.005 | | 787.76 | 0.147 | | 817.01 | 0.010 |  |
| **5** | **δAge + δAge^2^*Morph + μAge + μAge^2^*Morph + Laying date + δAge^2^*EL + δAge^2^*LL** | **655.43** | **0.340** | | **680.64** | **0.865** | | **786.13** | **0.332** | | 808.81 | 0.012 |  |
| 6 | δAge + δAge^2^*Morph + μAge + μAge^2^*Morph + Laying date + μAge^2^*EL + μAge^2^*LL | 656.96 | 0.158 | | 685.54 | 0.075 | | 787.44 | 0.172 | | 816.70 | 0.370 |  |
|  |  |  |  | |  |  | |  |  | |  |  |  |

**Table S4.** Marginal effects obtained from the *ggeffects* R package from the generalised mixed models exploring within-individual (δ) age effects on annual reproductive performance of female Eleonora’s falcons. Note that the probability column refers to the probability of producing 3 fledglings at a given age, which, for ease of interpretation has been transformed into predicted no. of fledglings. Key: D = dark, P = pale, SE = standard error, CI = 95% confidence interval.

| δ age | Predicted no. of fledglings | Predicted probability | SE | lower CI | upper CI | Morph |
| --- | --- | --- | --- | --- | --- | --- |
| -5.62 | 2 | 0.61377056 | 0.74911546 | 0.26794974 | 0.87340749 | P |
| -5 | 2 | 0.59857436 | 0.59324949 | 0.31794775 | 0.82667971 | P |
| -4.57 | 2 | 0.58911285 | 0.49845444 | 0.35054292 | 0.79203765 | P |
| -4.5 | 2 | 0.58765862 | 0.48409 | 0.35560202 | 0.78635516 | P |
| -4.38 | 2 | 0.58522245 | 0.4601769 | 0.36408432 | 0.77663787 | P |
| -3.57 | 2 | 0.57068825 | 0.32339476 | 0.41358072 | 0.71473675 | P |
| -3.5 | 2 | 0.56959076 | 0.31369311 | 0.41711072 | 0.70992274 | P |
| -3.33 | 2 | 0.5670318 | 0.29161357 | 0.42511727 | 0.69873961 | P |
| -3.17 | 2 | 0.5647616 | 0.27278551 | 0.43189101 | 0.68893811 | P |
| -3 | 2 | 0.56249707 | 0.25489345 | 0.43824703 | 0.6793714 | P |
| -2.8 | 2 | 0.5600284 | 0.23665868 | 0.44458726 | 0.66932192 | P |
| -2.62 | 2 | 0.557988 | 0.22283829 | 0.44923685 | 0.6614484 | P |
| -2.6 | 2 | 0.55777192 | 0.2214521 | 0.44969236 | 0.66064335 | P |
| -2.57 | 2 | 0.5574518 | 0.21942806 | 0.45035303 | 0.65946203 | P |
| -2.5 | 2 | 0.55672351 | 0.2149607 | 0.45179018 | 0.65682796 | P |
| -2.38 | 2 | 0.55553581 | 0.20811668 | 0.45392171 | 0.65270819 | P |
| -2.33 | 2 | 0.55506364 | 0.20556007 | 0.45468994 | 0.65113724 | P |
| -2.25 | 2 | 0.55433597 | 0.20181769 | 0.45577839 | 0.64879825 | P |
| -2.2 | 2 | 0.55389857 | 0.1996897 | 0.45637387 | 0.64744332 | P |
| -2.17 | 2 | 0.55364256 | 0.19848847 | 0.45670097 | 0.64666904 | P |
| -2 | 2 | 0.55228295 | 0.19269417 | 0.45815447 | 0.64280801 | P |
| -1.75 | 2 | 0.5505654 | 0.18694166 | 0.45922984 | 0.63861418 | P |
| -1.67 | 2 | 0.55008677 | 0.18568894 | 0.45935927 | 0.63760055 | P |
| -1.62 | 2 | 0.54980512 | 0.1850298 | 0.4593975 | 0.6370389 | P |
| -1.5 | 2 | 0.5491841 | 0.1837933 | 0.45937635 | 0.63589772 | P |
| -1.38 | 2 | 0.54864069 | 0.18297564 | 0.45922931 | 0.63501809 | P |
| -1.33 | 2 | 0.54843718 | 0.18273901 | 0.45914041 | 0.63472008 | P |
| -1.25 | 2 | 0.54813962 | 0.18246821 | 0.45897387 | 0.63431838 | P |
| -1.2 | 2 | 0.54797119 | 0.18235806 | 0.45885862 | 0.63411054 | P |
| -1.17 | 2 | 0.54787661 | 0.18231118 | 0.45878662 | 0.63400062 | P |
| -1 | 2 | 0.54743241 | 0.18226493 | 0.45836393 | 0.6335634 | P |
| -0.8 | 2 | 0.54710965 | 0.18252736 | 0.45791286 | 0.63338036 | P |
| -0.75 | 2 | 0.54706272 | 0.18262234 | 0.45781964 | 0.63337962 | P |
| -0.62 | 2 | 0.54700393 | 0.18289259 | 0.45762928 | 0.63344752 | P |
| -0.6 | 2 | 0.54700299 | 0.18293547 | 0.45760748 | 0.63346615 | P |
| -0.57 | 2 | 0.54700564 | 0.1829998 | 0.45757883 | 0.6334979 | P |
| -0.5 | 2 | 0.54703072 | 0.18314839 | 0.45753167 | 0.63358901 | P |
| -0.38 | 2 | 0.54713531 | 0.18339089 | 0.45751846 | 0.63379732 | P |
| -0.33 | 2 | 0.54720185 | 0.18348548 | 0.45753911 | 0.63390267 | P |
| -0.25 | 2 | 0.5473364 | 0.18362803 | 0.45760455 | 0.63409353 | P |
| -0.2 | 2 | 0.54743805 | 0.1837119 | 0.45766559 | 0.63422685 | P |
| -0.17 | 2 | 0.54750553 | 0.18376053 | 0.45770953 | 0.63431214 | P |
| 0 | 2 | 0.54797966 | 0.18402246 | 0.4580572 | 0.634875 | P |
| 0.2 | 2 | 0.54873715 | 0.18436169 | 0.45865147 | 0.6357377 | P |
| 0.25 | 2 | 0.54896024 | 0.18446685 | 0.458824 | 0.63599402 | P |
| 0.33 | 2 | 0.5493452 | 0.18466502 | 0.45911365 | 0.63644379 | P |
| 0.38 | 2 | 0.54960332 | 0.1848128 | 0.45930066 | 0.63675201 | P |
| 0.5 | 2 | 0.55027774 | 0.18526798 | 0.45975583 | 0.6375882 | P |
| 0.62 | 2 | 0.55102965 | 0.18591184 | 0.46019721 | 0.6385814 | P |
| 0.67 | 2 | 0.55136581 | 0.18625094 | 0.46036968 | 0.63904828 | P |
| 0.75 | 2 | 0.5519316 | 0.18689751 | 0.46062317 | 0.63986785 | P |
| 0.8 | 2 | 0.55230268 | 0.18737491 | 0.46076353 | 0.64042907 | P |
| 1 | 2 | 0.5539211 | 0.18997167 | 0.46112581 | 0.64310447 | P |
| 1.2 | 2 | 0.55575375 | 0.19395109 | 0.46103147 | 0.64658997 | P |
| 1.43 | 2 | 0.55812537 | 0.20073429 | 0.46011628 | 0.6518065 | P |
| 1.5 | 2 | 0.5589031 | 0.20334805 | 0.45962725 | 0.65368263 | P |
| 1.62 | 2 | 0.56029691 | 0.20849232 | 0.45852793 | 0.6572368 | P |
| 1.67 | 2 | 0.56090021 | 0.21089591 | 0.45796642 | 0.65884799 | P |
| 1.75 | 2 | 0.56189303 | 0.2150747 | 0.45693441 | 0.66158982 | P |
| 1.8 | 2 | 0.56253074 | 0.21790049 | 0.45620308 | 0.6634075 | P |
| 2 | 2 | 0.56521344 | 0.23092 | 0.45258126 | 0.67149247 | P |
| 2.2 | 2 | 0.56810636 | 0.24679665 | 0.44779514 | 0.68088499 | P |
| 2.25 | 2 | 0.56886229 | 0.25122378 | 0.44641193 | 0.68343445 | P |
| 2.38 | 2 | 0.57088873 | 0.26360026 | 0.44246376 | 0.69042887 | P |
| 2.4 | 2 | 0.57120829 | 0.26561555 | 0.44181129 | 0.69155082 | P |
| 2.5 | 2 | 0.57283722 | 0.27613661 | 0.43836965 | 0.69733814 | P |
| 2.62 | 2 | 0.57486019 | 0.28973678 | 0.43384886 | 0.70465881 | P |
| 2.67 | 2 | 0.575725 | 0.2957156 | 0.43184105 | 0.70782423 | P |
| 2.75 | 2 | 0.5771354 | 0.30566076 | 0.42847907 | 0.71302246 | P |
| 2.8 | 2 | 0.57803355 | 0.31211194 | 0.42628563 | 0.71635132 | P |
| 2.83 | 2 | 0.57857858 | 0.31606906 | 0.42493596 | 0.71837696 | P |
| 3 | 2 | 0.58175359 | 0.33970432 | 0.41682229 | 0.73022895 | P |
| 3.38 | 2 | 0.58937748 | 0.39979071 | 0.39599452 | 0.75859058 | P |
| 3.5 | 2 | 0.59193434 | 0.42078309 | 0.38870664 | 0.76793495 | P |
| 4.43 | 2 | 0.61409816 | 0.6143416 | 0.32311006 | 0.84139697 | P |
| 4.5 | 2 | 0.61592921 | 0.63103429 | 0.31767021 | 0.84672076 | P |
| 5 | 2 | 0.629635 | 0.75855087 | 0.27766351 | 0.8826102 | P |
| -5.62 | 0 | 0.02325244 | 2.26337589 | 0.00028182 | 0.66781455 | D |
| -5 | 0 | 0.05954167 | 1.74801476 | 0.00205436 | 0.66068649 | D |
| -4.57 | 0 | 0.10453451 | 1.42869707 | 0.00704742 | 0.65754397 | D |
| -4.5 | 0 | 0.11371163 | 1.3797305 | 0.00851311 | 0.65720095 | D |
| -4.38 | 0 | 0.13069568 | 1.29778386 | 0.01167651 | 0.65673728 | D |
| -3.57 | 1 | 0.28180019 | 0.81341941 | 0.07379379 | 0.65897351 | D |
| -3.5 | 1 | 0.29697922 | 0.77752894 | 0.08427329 | 0.65975541 | D |
| -3.33 | 1 | 0.33437912 | 0.69468836 | 0.11405147 | 0.66220128 | D |
| -3.17 | 1 | 0.36978562 | 0.62259592 | 0.14761859 | 0.66532795 | D |
| -3 | 1 | 0.4069988 | 0.55270543 | 0.18851797 | 0.66971505 | D |
| -2.8 | 1 | 0.44945439 | 0.48015919 | 0.24159219 | 0.67660634 | D |
| -2.62 | 1 | 0.48580575 | 0.42478107 | 0.29124443 | 0.6847663 | D |
| -2.6 | 1 | 0.48971394 | 0.41925301 | 0.29673989 | 0.68580311 | D |
| -2.57 | 1 | 0.49552319 | 0.41120524 | 0.30494592 | 0.68741027 | D |
| -2.5 | 2 | 0.50882236 | 0.39359019 | 0.3238511 | 0.6914092 | D |
| -2.38 | 2 | 0.53074509 | 0.36730546 | 0.35508307 | 0.69910534 | D |
| -2.33 | 2 | 0.53953843 | 0.35785352 | 0.3675143 | 0.70263237 | D |
| -2.25 | 2 | 0.55317583 | 0.34459896 | 0.38653737 | 0.70866566 | D |
| -2.2 | 2 | 0.56142429 | 0.33748668 | 0.39782947 | 0.71267392 | D |
| -2.17 | 2 | 0.56627059 | 0.33365027 | 0.40436953 | 0.71516306 | D |
| -2 | 2 | 0.59225943 | 0.31785936 | 0.43790245 | 0.73033179 | D |
| -1.75 | 2 | 0.62590806 | 0.31113279 | 0.47624112 | 0.7548245 | D |
| -1.67 | 2 | 0.63553971 | 0.31235818 | 0.48596523 | 0.76283176 | D |
| -1.62 | 2 | 0.64128512 | 0.31378164 | 0.49148722 | 0.767803 | D |
| -1.5 | 2 | 0.65422781 | 0.31888971 | 0.50316574 | 0.77948845 | D |
| -1.38 | 2 | 0.66600293 | 0.3258029 | 0.51289712 | 0.79062946 | D |
| -1.33 | 2 | 0.67057327 | 0.32905603 | 0.51645432 | 0.79506271 | D |
| -1.25 | 2 | 0.67748425 | 0.33456522 | 0.52161116 | 0.80186158 | D |
| -1.2 | 2 | 0.68155699 | 0.3381371 | 0.52453024 | 0.80591356 | D |
| -1.17 | 2 | 0.683911 | 0.34030913 | 0.52617862 | 0.80826839 | D |
| -1 | 2 | 0.69601196 | 0.3526679 | 0.53423687 | 0.82047882 | D |
| -0.8 | 2 | 0.70765773 | 0.36629481 | 0.54143243 | 0.83229213 | D |
| -0.75 | 2 | 0.71014904 | 0.36940139 | 0.54291777 | 0.83481187 | D |
| -0.62 | 2 | 0.71586657 | 0.37673166 | 0.54628445 | 0.84056397 | D |
| -0.6 | 2 | 0.71665039 | 0.37775372 | 0.54674384 | 0.84134774 | D |
| -0.57 | 2 | 0.71777891 | 0.37923007 | 0.54740557 | 0.84247345 | D |
| -0.5 | 2 | 0.72019373 | 0.38239986 | 0.5488271 | 0.84486914 | D |
| -0.38 | 2 | 0.72363266 | 0.38688556 | 0.55089097 | 0.84823632 | D |
| -0.33 | 2 | 0.72480812 | 0.38838371 | 0.55162049 | 0.84936847 | D |
| -0.25 | 2 | 0.72637799 | 0.39031029 | 0.55263645 | 0.85085427 | D |
| -0.2 | 2 | 0.72716638 | 0.39121497 | 0.55317957 | 0.85158165 | D |
| -0.17 | 2 | 0.72756866 | 0.39164593 | 0.55347219 | 0.85194443 | D |
| 0 | 2 | 0.72885247 | 0.39249022 | 0.55466601 | 0.8529684 | D |
| 0.2 | 2 | 0.7282057 | 0.39003203 | 0.55504828 | 0.85195114 | D |
| 0.25 | 2 | 0.7276791 | 0.38885206 | 0.55496275 | 0.85132295 | D |
| 0.33 | 2 | 0.72653092 | 0.38651506 | 0.55466484 | 0.850006 | D |
| 0.38 | 2 | 0.72562117 | 0.38478387 | 0.55437308 | 0.84898737 | D |
| 0.5 | 2 | 0.7228284 | 0.37983443 | 0.55331486 | 0.84592574 | D |
| 0.62 | 2 | 0.71916208 | 0.37387654 | 0.55169573 | 0.84198784 | D |
| 0.67 | 2 | 0.71737194 | 0.37113717 | 0.55083554 | 0.84008735 | D |
| 0.75 | 2 | 0.71418074 | 0.36649218 | 0.54920645 | 0.83672887 | D |
| 0.8 | 2 | 0.71197898 | 0.36345272 | 0.54801673 | 0.83443199 | D |
| 1 | 2 | 0.70153185 | 0.35071234 | 0.54170773 | 0.82375334 | D |
| 1.2 | 2 | 0.68834767 | 0.33833567 | 0.53227508 | 0.81084828 | D |
| 1.43 | 2 | 0.66958513 | 0.32749066 | 0.51610427 | 0.79383178 | D |
| 1.5 | 2 | 0.66307155 | 0.32552409 | 0.50974797 | 0.78835327 | D |
| 1.62 | 2 | 0.65099827 | 0.32423238 | 0.49698662 | 0.77883908 | D |
| 1.67 | 2 | 0.64562244 | 0.3246072 | 0.49090939 | 0.77487927 | D |
| 1.75 | 2 | 0.63659018 | 0.32649377 | 0.48018011 | 0.76861566 | D |
| 1.8 | 2 | 0.6306722 | 0.32854813 | 0.47281662 | 0.7647759 | D |
| 2 | 2 | 0.60486141 | 0.34433547 | 0.43803954 | 0.75038149 | D |
| 2.2 | 2 | 0.57557605 | 0.3734034 | 0.39479068 | 0.7381733 | D |
| 2.25 | 2 | 0.56771048 | 0.38282368 | 0.38277067 | 0.73552477 | D |
| 2.38 | 2 | 0.54625492 | 0.41130049 | 0.34964865 | 0.72942048 | D |
| 2.4 | 2 | 0.54282702 | 0.41618319 | 0.3443485 | 0.72858058 | D |
| 2.5 | 2 | 0.52519228 | 0.44254299 | 0.31723372 | 0.72476513 | D |
| 2.62 | 2 | 0.50297899 | 0.47830585 | 0.28382918 | 0.72099014 | D |
| 2.67 | 1 | 0.49340195 | 0.49447958 | 0.26981608 | 0.71965852 | D |
| 2.75 | 1 | 0.47770937 | 0.52184861 | 0.24749335 | 0.7178016 | D |
| 2.8 | 1 | 0.46768345 | 0.53985728 | 0.23369786 | 0.71680217 | D |
| 2.83 | 1 | 0.46159201 | 0.55098672 | 0.22550841 | 0.71625879 | D |
| 3 | 1 | 0.42611678 | 0.6184579 | 0.1809584 | 0.71390842 | D |
| 3.38 | 1 | 0.34301513 | 0.79408236 | 0.09918981 | 0.71228064 | D |
| 3.5 | 1 | 0.31649246 | 0.85606452 | 0.07960003 | 0.7125738 | D |
| 4.43 | 0 | 0.13655582 | 1.43039993 | 0.00949238 | 0.72298895 | D |
| 4.5 | 0 | 0.12615782 | 1.47996869 | 0.00787582 | 0.72418553 | D |
| 5 | 0 | 0.0673223 | 1.85858684 | 0.00188615 | 0.73384033 | D |

**Table S5.** Marginal effects obtained from the *ggeffects* R package from the generalised mixed models exploring between-individual (μ) age effects on annual reproductive performance of female and male Eleonora’s falcons. Note that the probability column refers to the probability of producing 3 fledglings at a given age, which, for ease of interpretation has been transformed into predicted no. of fledglings. Key: F = female, M = male, D = dark, P = pale, SE = standard error, CI = 95% confidence interval.

| μ age | Predicted no. of fledglings | Predicted probability | SE | lower CI | upper CI | Morph | Sex |
| --- | --- | --- | --- | --- | --- | --- | --- |
| 2 | 1 | 0.294957666 | 0.50950434 | 0.13353754 | 0.53175324 | D | F |
| 2.5 | 1 | 0.348947624 | 0.46276956 | 0.17789423 | 0.57036572 | D | F |
| 3 | 1 | 0.40707179 | 0.42264748 | 0.23068318 | 0.61118358 | D | F |
| 3.33 | 1 | 0.447006546 | 0.40016096 | 0.2695171 | 0.63911571 | D | F |
| 3.5 | 1 | 0.467876172 | 0.38997088 | 0.29049083 | 0.65377091 | D | F |
| 3.75 | 1 | 0.498742024 | 0.37690855 | 0.32218268 | 0.67561565 | D | F |
| 4 | 2 | 0.529606676 | 0.36639729 | 0.35444435 | 0.69776782 | D | F |
| 4.25 | 2 | 0.560235878 | 0.35876968 | 0.38673528 | 0.72016799 | D | F |
| 4.33 | 2 | 0.569950095 | 0.35699602 | 0.39698303 | 0.72737539 | D | F |
| 4.5 | 2 | 0.590402635 | 0.35438295 | 0.41849668 | 0.74273027 | D | F |
| 4.6 | 2 | 0.602291505 | 0.35361576 | 0.43093144 | 0.75177405 | D | F |
| 4.67 | 2 | 0.610542372 | 0.35343268 | 0.43951695 | 0.75810313 | D | F |
| 4.8 | 2 | 0.625693521 | 0.35389221 | 0.4551632 | 0.76983889 | D | F |
| 5 | 2 | 0.648515781 | 0.3567081 | 0.47836015 | 0.78779023 | D | F |
| 5.33 | 2 | 0.684682455 | 0.3672129 | 0.51390913 | 0.81684237 | D | F |
| 5.5 | 2 | 0.702501778 | 0.37555938 | 0.53075035 | 0.83136363 | D | F |
| 6 | 2 | 0.751342392 | 0.41174344 | 0.57414481 | 0.87133192 | D | F |
| 7 | 2 | 0.831788288 | 0.53307275 | 0.6349591 | 0.93358803 | D | F |
| 7.17 | 3 | 0.843172164 | 0.55958956 | 0.64227388 | 0.94151929 | D | F |
| 7.33 | 3 | 0.85330364 | 0.58598375 | 0.64845241 | 0.94830218 | D | F |
| 7.5 | 3 | 0.863465861 | 0.61551139 | 0.65430111 | 0.95481532 | D | F |
| 7.57 | 3 | 0.867474307 | 0.62810468 | 0.65650253 | 0.95729812 | D | F |
| 8 | 3 | 0.889950101 | 0.71085084 | 0.66751996 | 0.97021387 | D | F |
| 8.2 | 3 | 0.899214194 | 0.75240156 | 0.67124994 | 0.97499127 | D | F |
| 8.25 | 3 | 0.901419072 | 0.76308545 | 0.67205019 | 0.97607726 | D | F |
| 8.33 | 3 | 0.90485725 | 0.78042344 | 0.67322263 | 0.97773016 | D | F |
| 8.38 | 3 | 0.90695107 | 0.79141118 | 0.67388856 | 0.97871214 | D | F |
| 8.5 | 3 | 0.91180775 | 0.81825377 | 0.67527956 | 0.9809164 | D | F |
| 8.62 | 3 | 0.916433528 | 0.84575747 | 0.67638173 | 0.98291802 | D | F |
| 9 | 3 | 0.929655886 | 0.93714952 | 0.67800881 | 0.98808764 | D | F |
| 10.33 | 3 | 0.962079896 | 1.30692943 | 0.66196908 | 0.99696696 | D | F |
| 11.75 | 3 | 0.980708249 | 1.78516846 | 0.60580956 | 0.99940566 | D | F |
| 13 | 3 | 0.989431161 | 2.27663841 | 0.51926321 | 0.99987677 | D | F |
| 2 | 1 | 0.323713784 | 0.31249629 | 0.20599496 | 0.46897113 | P | F |
| 2.5 | 1 | 0.363384228 | 0.25913293 | 0.25567004 | 0.48679949 | P | F |
| 3 | 1 | 0.401129152 | 0.21813861 | 0.30400331 | 0.50669493 | P | F |
| 3.33 | 1 | 0.42457405 | 0.19834218 | 0.33341768 | 0.52116719 | P | F |
| 3.5 | 1 | 0.436119131 | 0.19043023 | 0.34747336 | 0.52904447 | P | F |
| 3.75 | 1 | 0.452382098 | 0.18153412 | 0.36659532 | 0.54109452 | P | F |
| 4 | 1 | 0.467744685 | 0.17568349 | 0.38378013 | 0.55357668 | P | F |
| 4.25 | 1 | 0.482163128 | 0.17251514 | 0.39903172 | 0.5662938 | P | F |
| 4.33 | 1 | 0.486571992 | 0.17199429 | 0.4035181 | 0.57037411 | P | F |
| 4.5 | 1 | 0.495604641 | 0.17156871 | 0.41244902 | 0.57900414 | P | F |
| 4.6 | 2 | 0.500702225 | 0.17169987 | 0.41733658 | 0.58402885 | P | F |
| 4.67 | 2 | 0.504174674 | 0.17193802 | 0.42060428 | 0.58751245 | P | F |
| 4.8 | 2 | 0.510412873 | 0.17265925 | 0.42635256 | 0.5938884 | P | F |
| 5 | 2 | 0.519472355 | 0.17434603 | 0.43443396 | 0.60339729 | P | F |
| 5.33 | 2 | 0.53298749 | 0.1781485 | 0.44595627 | 0.61805635 | P | F |
| 5.5 | 2 | 0.539251936 | 0.18038143 | 0.45110436 | 0.62501101 | P | F |
| 6 | 2 | 0.554933864 | 0.18713727 | 0.46352584 | 0.64277028 | P | F |
| 7 | 2 | 0.574171382 | 0.19878373 | 0.47733605 | 0.66563314 | P | F |
| 7.17 | 2 | 0.575856879 | 0.20057208 | 0.4781824 | 0.66794435 | P | F |
| 7.33 | 2 | 0.577026513 | 0.20229324 | 0.47853601 | 0.66975257 | P | F |
| 7.5 | 2 | 0.577827481 | 0.20422047 | 0.47841256 | 0.67131223 | P | F |
| 7.57 | 2 | 0.578025166 | 0.20505932 | 0.47820453 | 0.6718536 | P | F |
| 8 | 2 | 0.577549915 | 0.21118156 | 0.47472539 | 0.67406572 | P | F |
| 8.2 | 2 | 0.576337974 | 0.21486262 | 0.47168912 | 0.67455971 | P | F |
| 8.25 | 2 | 0.575936494 | 0.21589278 | 0.47077637 | 0.67464204 | P | F |
| 8.33 | 2 | 0.575212053 | 0.21764387 | 0.46918274 | 0.67474444 | P | F |
| 8.38 | 2 | 0.574707932 | 0.21880647 | 0.46810164 | 0.6747918 | P | F |
| 8.5 | 2 | 0.573336648 | 0.22182756 | 0.46523213 | 0.67486053 | P | F |
| 8.62 | 2 | 0.57173707 | 0.225202 | 0.46196216 | 0.67487762 | P | F |
| 9 | 2 | 0.565158316 | 0.23858781 | 0.44880513 | 0.67474984 | P | F |
| 10.33 | 2 | 0.523730072 | 0.32717816 | 0.36673121 | 0.67617509 | P | F |
| 11.75 | 1 | 0.44787647 | 0.50219877 | 0.23262536 | 0.68460952 | P | F |
| 13 | 1 | 0.357875296 | 0.72085307 | 0.11947155 | 0.69598438 | P | F |
| 2 | 1 | 0.330446719 | 0.3910351 | 0.18655078 | 0.5150578 | NA | M |
| 2.5 | 1 | 0.363339312 | 0.32568052 | 0.23161368 | 0.51934594 | NA | M |
| 3 | 1 | 0.394602055 | 0.27355474 | 0.27604494 | 0.52701099 | NA | M |
| 3.33 | 1 | 0.414091093 | 0.24687925 | 0.30344365 | 0.53414579 | NA | M |
| 3.5 | 1 | 0.423727321 | 0.23559463 | 0.31664184 | 0.53849024 | NA | M |
| 4 | 1 | 0.450327504 | 0.21184357 | 0.35102123 | 0.55375599 | NA | M |
| 4.25 | 1 | 0.46259099 | 0.20483621 | 0.36554249 | 0.5625593 | NA | M |
| 4.33 | 1 | 0.466363005 | 0.20319654 | 0.36981526 | 0.56549805 | NA | M |
| 4.5 | 1 | 0.474128533 | 0.20057815 | 0.37831548 | 0.57188537 | NA | M |
| 4.6 | 1 | 0.478535841 | 0.19953751 | 0.38296178 | 0.57570756 | NA | M |
| 4.67 | 1 | 0.481549462 | 0.19900694 | 0.38606529 | 0.57840051 | NA | M |
| 4.86 | 1 | 0.489429919 | 0.19828888 | 0.39390531 | 0.58573278 | NA | M |
| 5 | 1 | 0.494954341 | 0.19833335 | 0.39917362 | 0.59110685 | NA | M |
| 5.33 | 2 | 0.507019258 | 0.19983392 | 0.41009119 | 0.60342251 | NA | M |
| 5.5 | 2 | 0.512706668 | 0.20112103 | 0.41499538 | 0.60945635 | NA | M |
| 5.67 | 2 | 0.518033653 | 0.20260552 | 0.41947386 | 0.61520988 | NA | M |
| 5.75 | 2 | 0.520415592 | 0.2033451 | 0.42144574 | 0.61780853 | NA | M |
| 6 | 2 | 0.527343803 | 0.20571688 | 0.42709505 | 0.62543502 | NA | M |
| 6.5 | 2 | 0.538860758 | 0.21019377 | 0.43629436 | 0.63823908 | NA | M |
| 6.6 | 2 | 0.540790844 | 0.21099778 | 0.43781827 | 0.64039405 | NA | M |
| 6.75 | 2 | 0.54345341 | 0.2121424 | 0.43990731 | 0.6433754 | NA | M |
| 7 | 2 | 0.54727233 | 0.21391684 | 0.4428475 | 0.64769556 | NA | M |
| 7.25 | 2 | 0.550320264 | 0.21561876 | 0.44506264 | 0.65125716 | NA | M |
| 7.33 | 2 | 0.551133192 | 0.21617377 | 0.4456055 | 0.65224978 | NA | M |
| 7.5 | 2 | 0.552599727 | 0.21741669 | 0.44646879 | 0.65414494 | NA | M |
| 7.6 | 2 | 0.553296822 | 0.21821279 | 0.44678013 | 0.65513527 | NA | M |
| 7.67 | 2 | 0.553711889 | 0.21881031 | 0.44690578 | 0.65577903 | NA | M |
| 8 | 2 | 0.554860901 | 0.22228951 | 0.4463699 | 0.65836354 | NA | M |
| 8.67 | 2 | 0.553097047 | 0.23543125 | 0.43825557 | 0.66253885 | NA | M |
| 8.86 | 2 | 0.551596615 | 0.24143121 | 0.43387149 | 0.66381017 | NA | M |
| 9 | 2 | 0.550207487 | 0.2466788 | 0.42997026 | 0.66485164 | NA | M |
| 9.14 | 2 | 0.548577343 | 0.2526928 | 0.42547325 | 0.66600986 | NA | M |
| 9.33 | 2 | 0.545978723 | 0.26217194 | 0.41838379 | 0.6678071 | NA | M |
| 9.5 | 2 | 0.543275726 | 0.27201916 | 0.41105382 | 0.66966822 | NA | M |
| 10.33 | 2 | 0.524925345 | 0.34016729 | 0.36194515 | 0.68276323 | NA | M |
| 11.67 | 1 | 0.477203336 | 0.52139568 | 0.24728099 | 0.71721055 | NA | M |

**Table S6.** Marginal effects obtained from the *ggeffects* R package from the generalised mixed models exploring late-life environmental effects on annual reproductive performance of female and male Eleonora’s falcons. Note that the probability column refers to the probability of producing 3 fledglings at a given age, which, for ease of interpretation has been transformed into predicted no. of fledglings and also note that not all predictions are reported but a subsample along the mean u-wind component. Key: F = female, M = male, D = dark, P = pale, SE = standard error, CI = 95% confidence interval.

| Wind | Predicted no. of fledglings | Predicted  probability | Lower, upper CI | Sex |
| --- | --- | --- | --- | --- |
| -2.44 | 3 | 0.78 | 0.65, 0.87 | F |
| -2.16 | 2 | 0.75 | 0.63, 0.84 | F |
| -1.12 | 2 | 0.66 | 0.56, 0.74 | F |
| -0.76 | 2 | 0.62 | 0.52, 0.70 | F |
| -0.45 | 2 | 0.58 | 0.49, 0.67 | F |
| -0.28 | 2 | 0.57 | 0.48, 0.65 | F |
| 0.41 | 1 | 0.49 | 0.40, 0.58 | F |
| 0.72 | 1 | 0.45 | 0.36, 0.55 | F |
| 1.08 | 1 | 0.41 | 0.31, 0.52 | F |
| 3.52 | 0 | 0.19 | 0.09, 0.36 | F |
| -2.38 | 3 | 0.81 | 0.69, 0.89 | M |
| -2.10 | 3 | 0.78 | 0.66, 0.86 | M |
| -1.00 | 2 | 0.66 | 0.55, 0.75 | M |
| -0.70 | 2 | 0.62 | 0.52, 0.71 | M |
| -0.22 | 2 | 0.55 | 0.45, 0.65 | M |
| 0.78 | 2 | 0.42 | 0.31, 0.53 | M |
| 1.10 | 1 | 0.37 | 0.27, 0.49 | M |
| 3.58 | 1 | 0.13 | 0.06, 0.26 | M |

**Table S7.** Marginal effects obtained from the *ggeffects* R package from the generalised mixed model exploring early-life environmental effects on lifetime reproductive performance (LRS) of female and male Eleonora’s falcons. Note that not all predictions are reported but a subsample along the mean u-wind component (EL environment) and observed lifespan. Key: SE = standard error, CI = 95% confidence interval.

| El environment | Predicted LRS | SE | Lower CI | Upper CI | Lifespan (years) |
| --- | --- | --- | --- | --- | --- |
| -3.2327403 | 1.44 | 0.17 | 1.17 | 2.29 | 3.5 |
| -2.9548139 | 1.53 | 0.15 | 1.30 | 2.33 | 3.5 |
| -1.9109497 | 1.93 | 0.09 | 1.84 | 2.60 | 3.5 |
| -1.8507248 | 1.95 | 0.09 | 1.87 | 2.63 | 3.5 |
| -1.5522809 | 2.08 | 0.08 | 2.01 | 2.79 | 3.5 |
| -1.4025054 | 2.15 | 0.08 | 2.07 | 2.89 | 3.5 |
| -1.2450071 | 2.23 | 0.09 | 2.13 | 3.01 | 3.5 |
| -1.2450071 | 2.23 | 0.09 | 2.13 | 3.01 | 3.5 |
| -1.0756662 | 2.31 | 0.09 | 2.18 | 3.16 | 3.5 |
| -0.3877632 | 2.69 | 0.14 | 2.34 | 3.98 | 3.5 |
| -0.0624614 | 2.89 | 0.16 | 2.40 | 4.48 | 3.5 |
| 0.29090837 | 3.12 | 0.19 | 2.46 | 5.11 | 3.5 |
| 0.60649199 | 3.34 | 0.21 | 2.50 | 5.76 | 3.5 |
| -3.2327403 | 3.57 | 0.12 | 3.21 | 5.11 | 6.4 |
| -2.9548139 | 3.63 | 0.11 | 3.35 | 5.08 | 6.4 |
| -1.9109497 | 3.89 | 0.07 | 3.85 | 5.07 | 6.4 |
| -1.8507248 | 3.91 | 0.07 | 3.87 | 5.08 | 6.4 |
| -1.5522809 | 3.98 | 0.07 | 3.97 | 5.16 | 6.4 |
| -1.4025054 | 4.02 | 0.07 | 4.01 | 5.21 | 6.4 |
| -1.2450071 | 4.07 | 0.07 | 4.04 | 5.28 | 6.4 |
| -1.2450071 | 4.07 | 0.07 | 4.04 | 5.28 | 6.4 |
| -1.0756662 | 4.11 | 0.07 | 4.07 | 5.36 | 6.4 |
| -0.3877632 | 4.30 | 0.09 | 4.08 | 5.85 | 6.4 |
| -0.0624614 | 4.40 | 0.11 | 4.06 | 6.13 | 6.4 |
| 0.29090837 | 4.50 | 0.12 | 4.03 | 6.48 | 6.4 |
| 0.60649199 | 4.59 | 0.14 | 3.99 | 6.82 | 6.4 |
| -3.2327403 | 8.81 | 0.18 | 7.04 | 14.23 | 9.3 |
| -2.9548139 | 8.60 | 0.16 | 7.14 | 13.37 | 9.3 |
| -1.9109497 | 7.85 | 0.09 | 7.41 | 10.74 | 9.3 |
| -1.8507248 | 7.81 | 0.09 | 7.42 | 10.62 | 9.3 |
| -1.5522809 | 7.61 | 0.08 | 7.41 | 10.09 | 9.3 |
| -1.4025054 | 7.52 | 0.07 | 7.38 | 9.87 | 9.3 |
| -1.2450071 | 7.41 | 0.07 | 7.33 | 9.68 | 9.3 |
| -1.2450071 | 7.41 | 0.07 | 7.33 | 9.68 | 9.3 |
| -1.0756662 | 7.31 | 0.07 | 7.23 | 9.52 | 9.3 |
| -0.3877632 | 6.88 | 0.09 | 6.57 | 9.29 | 9.3 |
| -0.0624614 | 6.69 | 0.11 | 6.18 | 9.34 | 9.3 |
| 0.29090837 | 6.49 | 0.13 | 5.74 | 9.46 | 9.3 |
| 0.60649199 | 6.31 | 0.15 | 5.36 | 9.59 | 9.3 |

**Table S8.** Individual sample sizes and number of breeding attempts monitored per individual in Eleonora’s falcons in this study. Below, the age classes and number of individuals per age class are shown.

| **No. of monitored breeding attempts (sampling occasions)** | **1** | **2** | **3** | **4** | **5** | **6** | **7** | **8** | **9** | **10** | **-** | **-** | **-** |
| --- | --- | --- | --- | --- | --- | --- | --- | --- | --- | --- | --- | --- | --- |
| *Total N females* | *38* | *27* | *14* | *10* | *6* | *5* | *2* | *1* | *-* | *-* | *-* | *-* | *-* |
| N dark females | 4 | 2 | 1 | - | 2 | - | - | 1 | - | - | - | - | - |
| N pale females | 34 | 25 | 13 | 10 | 4 | 5 | 2 | - | - | - | - | - | - |
|  |  |  |  |  |  |  |  |  |  |  |  |  |  |
| *Total N males* | *39* | *39* | *20* | *8* | *6* | *6* | *2* | *-* | *1* | *1* | *-* | *-* | *-* |
| N dark males | 4 | 7 | 5 | 5 | 2 | 3 | - | - | - | - | - | - | - |
| N pale males | 35 | 32 | 15 | 3 | 4 | 3 | 2 | - | 1 | 1 |  |  |  |
|  |  |  |  |  |  |  |  |  |  |  |  |  |  |
| **Age (years)** | **2** | **3** | **4** | **5** | **6** | **7** | **8** | **9** | **10** | **11** | **12** | **13** | **14** |
| N dark females | 2 | 6 | 6 | 4 | 3 | 2 | 2 | 2 | - | 1 | - | 1 | - |
| N pale females | 20 | 36 | 41 | 31 | 31 | 19 | 12 | 15 | 10 | 4 | 6 | - | 2 |
|  |  |  |  |  |  |  |  |  |  |  |  |  |  |
| N dark males | 4 | 11 | 14 | 13 | 14 | 10 | 6 | 6 | 3 | - | - | - | - |
| N pale males | 9 | 40 | 36 | 43 | 22 | 21 | 18 | 16 | 12 | 6 | 7 | 4 | - |

**Table S9:** Fixed effects that were included in the Bayesian model selection approach to evaluate early- and late-life environmental, age and morph effects on annual reproductive performance of male and female Eleonora’s falcons. Key: LL = late-life environmental conditions (productivity/wind intensity of breeding year); EL = early-life environmental conditions (productivity/wind intensity of year of birth); δAge = delta age, representing within-individual age effects; μAge = average age, representing between-individual age effects; ELPD diff = Expected Log pointwise Predictive Density difference; SE diff = standard error difference. Selected models are highlighted in bold (model 6 for females and model 7 for males).

|  |  |  |  |  | | |  |  |  | |  |  |  |
| --- | --- | --- | --- | --- | --- | --- | --- | --- | --- | --- | --- | --- | --- |
|  |  | **Females’ models** | | | | | | **Males’ models** | | | | |  |
|  |  | Productivity  EL and LL | | | Wind  EL and LL | | | Productivity  EL and LL | | Wind  EL and LL | | |  |
| *Model no.* | *Predictors* | *ELPD diff* | *SE diff* | | *ELPD diff* | *SE diff* | | *ELPD diff* | *SE diff* | | *ELPD diff* | *SE diff* |  |
| 1 | Age*Morph + Age^2^*Morph +  Laying date + LL env | -2.3 | 5.3 | | -0.5 | 4.3 | | 0 | 0 | | 0 | 0 |  |
| 2 | δAge*Morph + δAge^2^*Morph + μAge*Morph + μAge^2^*Morph + Laying date + LL*EL | -3.4 | 4.7 | | -1.4 | 2.9 | | -6.5 | 2.7 | | -6.3 | 2.8 |  |
| 3 | δAge*Morph + δAge^2^*Morph + μAge*Morph + μAge^2^*Morph + Laying date + δAge^2^*LL*EL | -7.8 | -4.6 | | -4.8 | 2.9 | | -11.0 | 2.9 | | -8.1 | 3.3 |  |
| 4 | δAge*Morph + δAge^2^*Morph + μAge*Morph + μAge^2^*Morph + Laying date + μAge^2^*LL*EL | -0.7 | 2.2 | | -1.4 | 1.6 | | -6.4 | 3.7 | | -8.5 | 3.0 |  |
| 5 | δAge + δAge^2^*Morph + μAge + μAge^2^*Morph + Laying date + δAge^2^*LL*EL | -6.5 | 4.0 | | -3.5 | 2.5 | | -9.0 | 3.0 | | -5.6 | 3.5 |  |
| 6 | **δAge + δAge^2^*Morph + μAge + μAge^2^*Morph + Laying date + μAge^2^*LL*EL** | **0** | **0** | | **0** | **0** | | -3.8 | 3.7 | | -4.2 | 3.3 |  |
| 7 | **δAge + δAge^2^ + μAge +**  **μAge^2^*LL*EL + Morph + Laying date** | -4.2 | 3.7 | | -4.3 | 3.6 | | **-1.8** | **3.7** | | **-3.7** | **3.2** |  |
|  |  |  |  | |  |  | |  |  | |  |  |  |

**Table S10.** Parameter estimates from the Bayesian models explaining age, morph and environmental effects on annual reproductive performance of female and male Eleonora’s falcons. Environmental conditions here were characterised using the mean annual productivity. β = parameter estimate, SE = standard error, σ2 = residual variance, τ = variance, ICC = interclass correlation coefficient, CI = confidence interval, LL = late-life, EL = early-life. Laying date, EL environment and LL environment were scaled. The baseline level for morph was the pale morph. Note that parameter estimates represent the odds ratio of producing an increasing number of fledglings per breeding attempt and thus, a significant relationship appears when CI do not overlap 1 (in bold).

|  |  | | | |  | | | |  |
| --- | --- | --- | --- | --- | --- | --- | --- | --- | --- |
|  | **Females model** | | | | **Males model** | | | |  |
| *Predictors* | β | *SE* | *CI (95%)* | β | | | *SE* | *CI (95%)* |  |
| Intercept 1 | 2.28 | 2.30 | 0.23 – 24.33 | 1.05 | | | 1.12 | 0.08 – 12.83 |  |
| Intercept 2 | **14.73** | **14.95** | **1.43 – 167.08** | 6.66 | | | 7.07 | 0.52 – 83.42 |  |
| Intercept 3 | **337.01** | **351.44** | **29.94 – 4360.14** | **103.06** | | | **109.75** | **8.19 – 1400.08** |  |
| **δAge** | 1.06 | 0.10 | 0.89 – 1.28 | **1.21** | | | **0.10** | **1.03 – 1.44** |  |
| δAge^2^ | 0.97 | 0.03 | 0.92 – 1.03 | 1.02 | | | 0.03 | 0.96 – 1.09 |  |
| **μAge** | **2.32** | **0.83** | **1.13 – 4.88** | 1.53 | | | 0.60 | 0.69 – 3.38 |  |
| μAge^2^ | 1.06 | 0.10 | 0.89 – 1.28 | 0.98 | | | 0.03 | 0.92 – 1.05 |  |
| Morph | 1.12 | 0.47 | 0.50 – 2.64 | 1.26 | | | 0.19 | 0.94 – 1.70 |  |
| **Morph*μAge^2^** | **0.98** | **0.01** | **0.95 – 0.99** | - | | | - | - |  |
| **Morph*δAge^2^** | **1.15** | **0.07** | **1.03 – 1.29** | - | | | - | - |  |
| **Laying date** | **0.71** | **0.12** | **0.50 – 0.99** | 0.85 | | | 0.11 | 0.66 – 1.09 |  |
| **LL environment** | **7.45** | **4.50** | **2.17 – 30.42** | **7.21** | | | **4.71** | **1.93 – 30.50** |  |
| **EL environment** | **0.11** | **0.11** | **0.01 – 0.99** | **0.10** | | | **0.08** | **0.01 – 0.59** |  |
| EL environment*LL environment | 0.09 | 0.12 | 0.00 – 4.16 | 0.55 | | | 0.74 | 0.01 – 32.14 |  |
| μAge^2^*LL environment | 1.02 | 0.01 | 1.00 – 1.05 | 1.02 | | | 0.01 | 0.99 – 1.05 |  |
| **μAge^2^*EL environment** | **1.06** | **0.03** | **1.01 – 1.13** | **1.05** | | | **0.02** | **1.01 – 1.09** |  |
| μAge^2^*LL environment*LL environment | 1.10 | 0.05 | 1.00 – 1.21 | 1.04 | | | 0.05 | 0.95 – 1.14 |  |
| *Random Effects* |  | | | | | | | |  |
| σ^2^ | 0.01 | | | | | 0.00 | | |  |
| τ_00_ | 0.90 | | | | | 0.91 | | |  |
| ICC | 0.01 | | | | | 0.00 | | |  |
| N _Nest site_ | 8 | | | | | 9 | | | |
| N _Birth year_ | 11 | | | | | 12 | | | |
| N _Breeding year_ | 13 | | | | | 12 | | | |
| N _ID: Birth year_ | 103 | | | | | 123 | | | |
| Observations | 256 | | | | | 308 | | |  |
| Marginal R^2^ / Conditional R^2^ | 0.391 / 0.432 | | | | | 0.355 / 0.387 | | |  |

**Table S11.** Parameter estimates from the Bayesian models explaining age, morph and environmental effects on annual reproductive performance of female and male Eleonora’s falcons. Environmental conditions here were characterised using the mean annual wind intensity. β = parameter estimate, SE = standard error, σ^2^ = residual variance, τ = variance, ICC = interclass correlation coefficient, CI = confidence interval, LL = late-life, EL = early-life. Laying date, EL environment and LL environment were scaled. Note that parameter estimates represent the odds ratio of producing an increasing number of fledglings per breeding attempt and thus a significant relationship appears when CI do not overlap 1 (marginally non-significant and significant relationships are highlighted in bold).

|  | **Females model** | | | | **Males model** | | | |  |
| --- | --- | --- | --- | --- | --- | --- | --- | --- | --- |
| *Predictors* | β | *SE* | *CI (95%)* | β | | | *SE* | *CI (95%)* |  |
| Intercept 1 | 1.17 | 1.23 | 0.10 – 14.33 | 0.97 | | | 1.06 | 0.07 – 13.56 |  |
| Intercept 1 | 7.37 | 7.79 | 0.63 – 95.53 | 6.33 | | | 6.90 | 0.48 – 92.17 |  |
| Intercept 3 | **152.88** | **165.10** | **12.23 – 2181.99** | **92.73** | | | **102.03** | **6.90 – 1420.28** |  |
| **δAge** | 1.04 | 0.11 | 0.86 – 1.28 | **1.19** | | | **0.11** | **1.00 – 1.42** |  |
| δAge^2^ | 1.02 | 0.04 | 0.95 – 1.10 | 1.05 | | | 0.04 | 0.98 – 1.12 |  |
| μAge | 1.70 | 0.72 | 0.73 – 3.99 | 1.69 | | | 0.69 | 0.75 – 3.78 |  |
| μAge^2^ | 0.98 | 0.04 | 0.91 – 1.05 | 0.97 | | | 0.03 | 0.91 – 1.03 |  |
| Morph | 0.71 | 0.54 | 0.13 – 3.42 | 0.67 | | | 0.20 | 0.37 – 1.21 |  |
| **Morph*μAge^2^** | **1.06** | **0.02** | **1.01 – 1.11** | - | | | - | - |  |
| **Morph*δAge^2^** | **0.76** | **0.09** | **0.60 – 0.95** | - | | | - | - |  |
| **Laying date** | **0.70** | **0.12** | **0.50 – 0.99** | 0.83 | | | 0.10 | 0.64 – 1.07 |  |
| **LL environment** | 0.62 | 0.19 | 0.32 – 1.16 | **0.52** | | | **0.14** | **0.29 – 0.90** |  |
| EL environment | 1.49 | 0.50 | 0.79 – 3.20 | 1.34 | | | 0.44 | 0.70 – 2.64 |  |
| EL environment*LL environment | 0.71 | 0.16 | 0.44 – 1.12 | 1.09 | | | 0.31 | 0.62 – 1.93 |  |
| **μAge^2^*LL environment** | **0.99** | **0.01** | **0.98 – 1.00** | **0.99** | | | **0.01** | **0.98 – 1.00** |  |
| **μAge^2^*EL environment** | **0.99** | **0.01** | **0.97 – 1.00** | 0.99 | | | 0.01 | 0.98 – 1.01 |  |
| **μAge^2^*LL environment*LL environment** | **1.01** | **0.01** | **1.00 – 1.02** | 1.00 | | | 0.01 | 0.99 – 1.02 |  |
| *Random Effects* |  | | | | | | | |  |
| σ^2^ | 0.02 | | | | | 0.02 | | |  |
| τ_00_ | 0.88 | | | | | 0.90 | | |  |
| ICC | 0.03 | | | | | 0.02 | | |  |
| N _Nest site_ | 8 | | | | | 9 | | | |
| N _Birth year_ | 11 | | | | | 12 | | | |
| N _Breeding year_ | 13 | | | | | 12 | | | |
| N _ID: Birth year_ | 103 | | | | | 123 | | | |
| Observations | 256 | | | | | 308 | | |  |
| Marginal R^2^ / Conditional R^2^ | 0.343 / 0.413 | | | | | 0.325 / 0.382 | | |  |

**Table S12.** Parameter estimates (bootstrapped) and random effects from the generalised mixed model explaining the effect of lifespan, morph and early-life environmental conditions on lifetime reproductive performance (censored to individuals born before 2016) of female and male Eleonora’s falcons. β = parameter estimate, SE = standard error, σ^2^ = residual variance, τ = variance, ICC = interclass correlation coefficient, EL = early-life. The baseline level for morph was the pale morph. Morph and sex were mean centred and EL environment was standardised. Note that parameter estimates represent the logarithmic odds ratio of producing an increasing number of fledglings throughout an individual’s lifetime. Significant predictors are highlighted in bold.

|  |  | | | |
| --- | --- | --- | --- | --- |
| *Predictors* | β | *SE* | *Z* | *P - value* |
| (Intercept) | 1.53 | 0.07 | 21.93 | **<0.001** |
| EL environment | 0.09 | 0.06 | 1.47 | 0.142 |
| **Morph** | **0.25** | **0.13** | **1.97** | **0.049** |
| Sex | -0.03 | 0.10 | -0.31 | 0.754 |
| **Observed lifespan** | **0.56** | **0.05** | **10.63** | **<0.001** |
| EL environment × Morph | 0.17 | 0.12 | 1.36 | 0.174 |
| EL environment × Sex | 0.01 | 0.10 | 0.12 | 0.907 |
| Morph × Sex | 0.23 | 0.24 | 0.99 | 0.323 |
| **EL environment × Observed lifespan** | **-0.15** | **0.06** | **-2.50** | **0.012** |
| Sex × Observed lifespan | 0.18 | 0.10 | 1.81 | 0.070 |
| EL environment × Morph × Sex | 0.01 | 0.24 | 0.06 | 0.955 |
| EL environment × Sex × Observed lifespan | -0.04 | 0.11 | -0.38 | 0.706 |
| **Random Effects** | | | | |
| σ^2^ | 0.23 | | | |
| τ _birth year_ | 0.01 | | | |
| ICC | 0.04 | | | |
| N _birth year_ | 7 | | | |
| Observations | 135 | | | |
| Marginal R^2^ / Conditional R^2^ | 0.595 / 0.610 | | | |

**Figure S1:** Annual offspring (number of fledglings) in relation to laying date in female Eleonora’s falcons. The regression line represents the smoothed predicted values from the model with grey shaded area representing the upper and lower 95% confidence intervals. Raw data points are also shown.

**
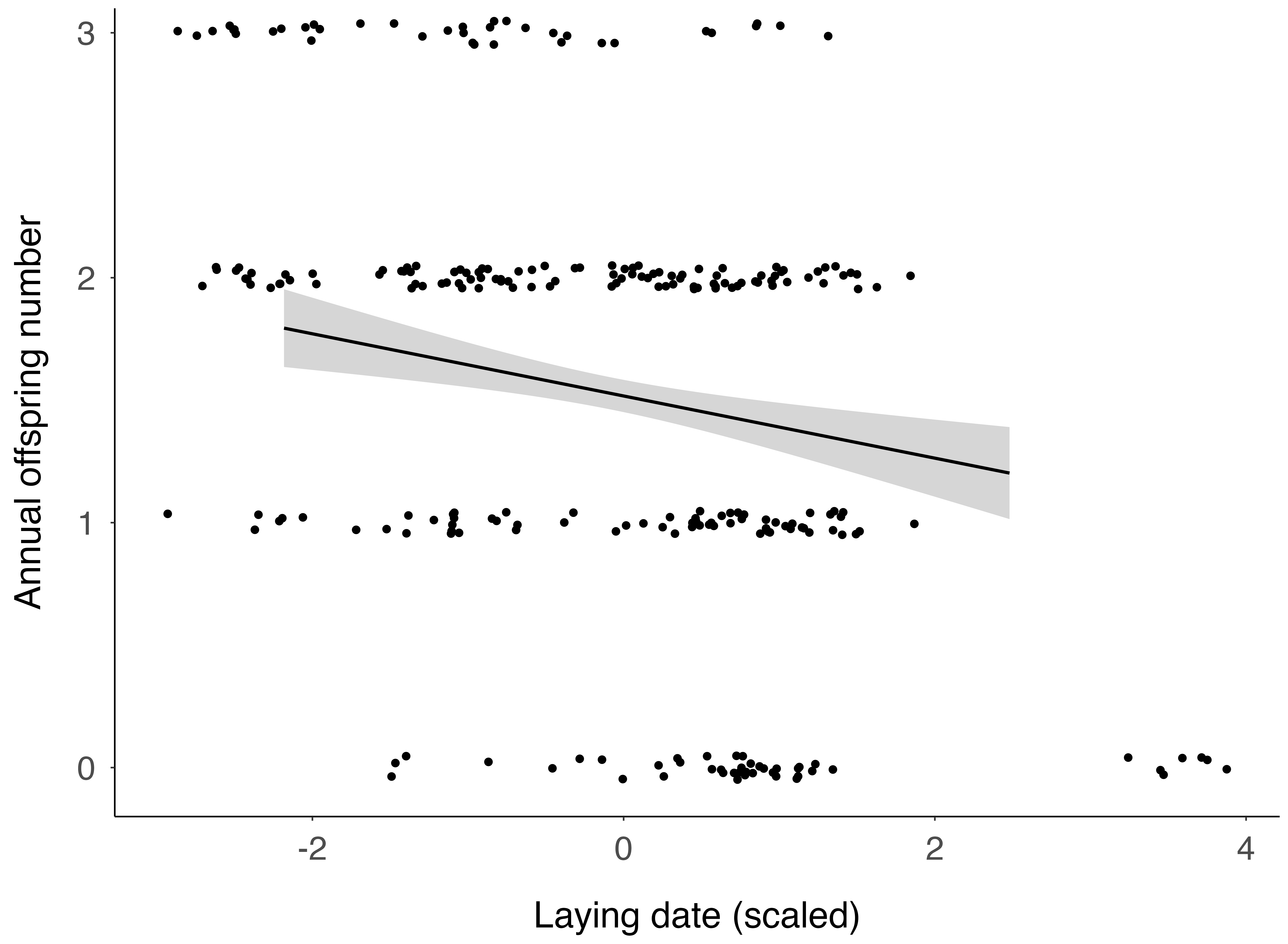
**

**Figure S2:** Annual offspring (number of fledglings) in relation to mean annual wind intensity of the breeding year for female (a) and for male (b) Eleonora’s falcons; and in relation to mean annual productivity for females (c) and for males (d). Lines and shaded areas represent the predicted values from the model and upper and lower 95% confidence intervals. Raw data points are also shown.

**(a)**
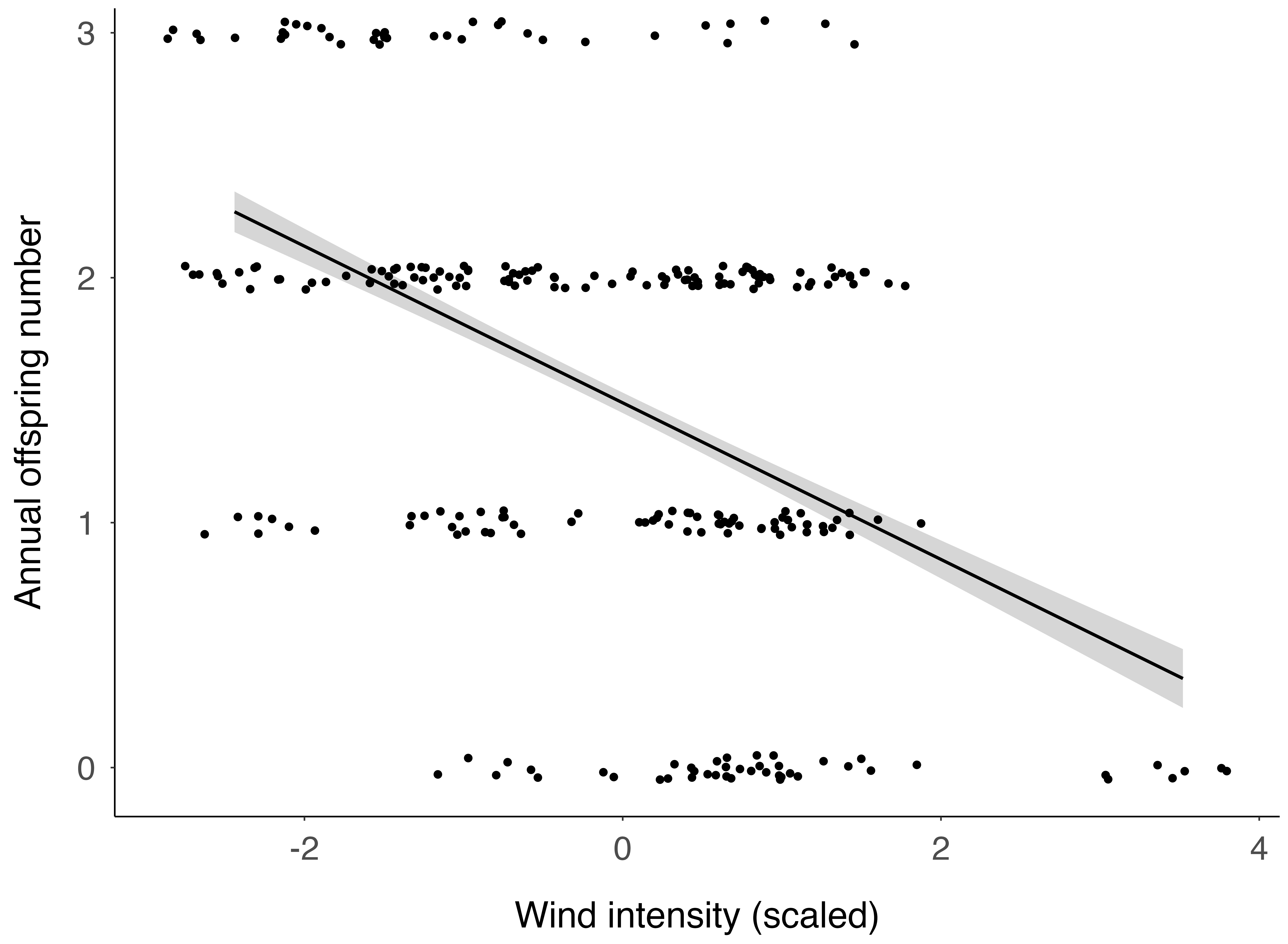


**(c)**

**
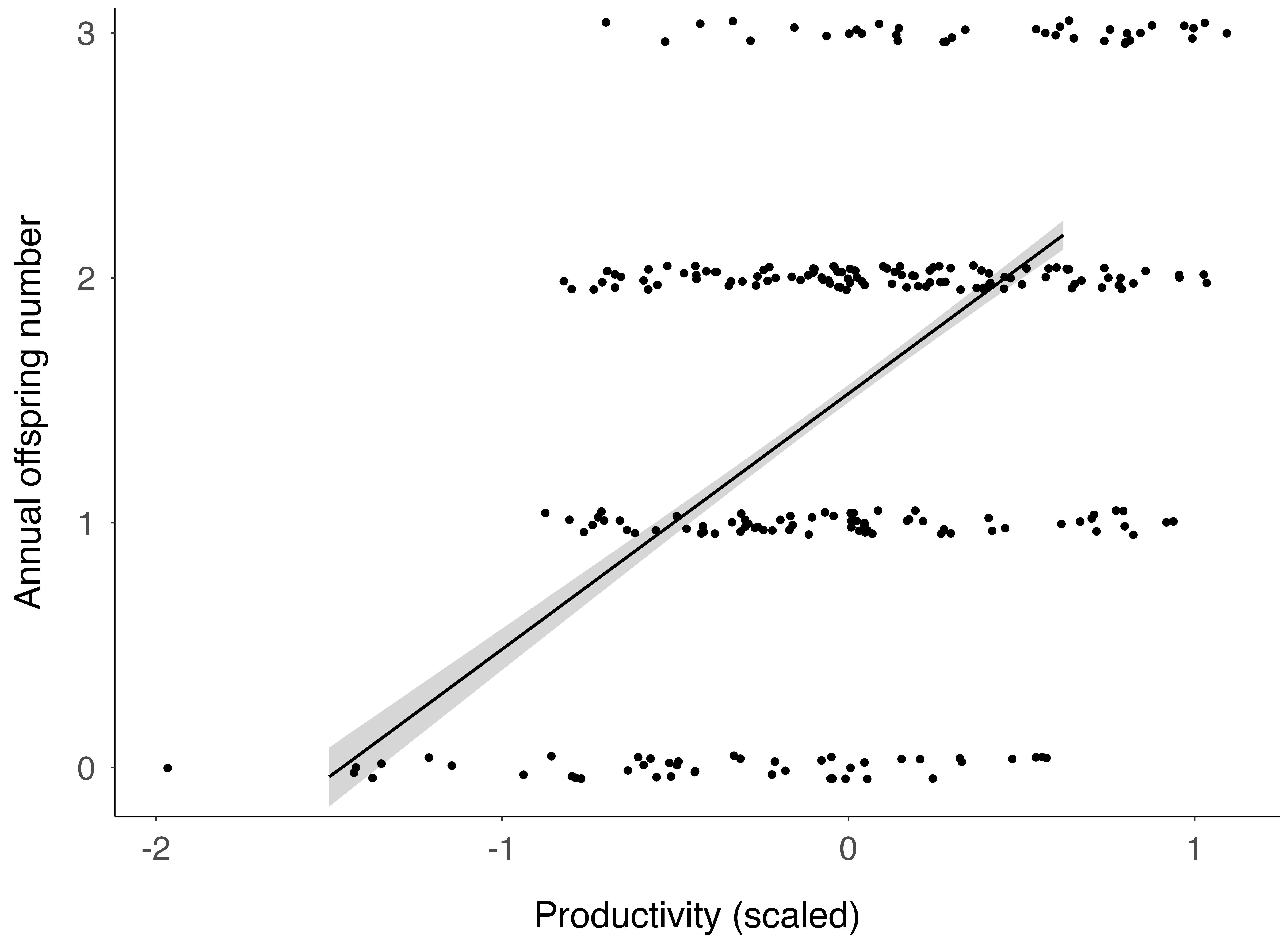
**

**(b)
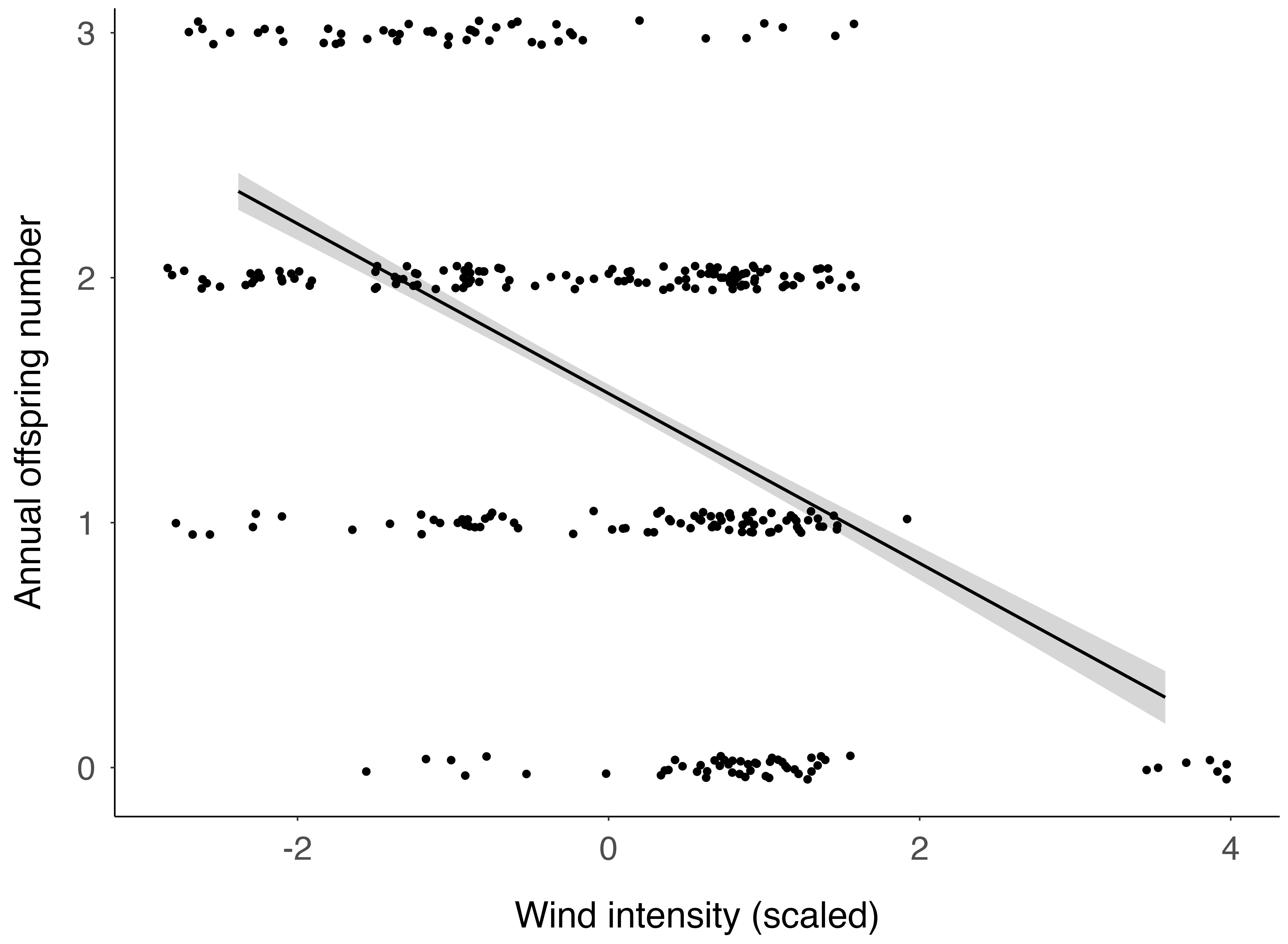
**

**(d)**

**
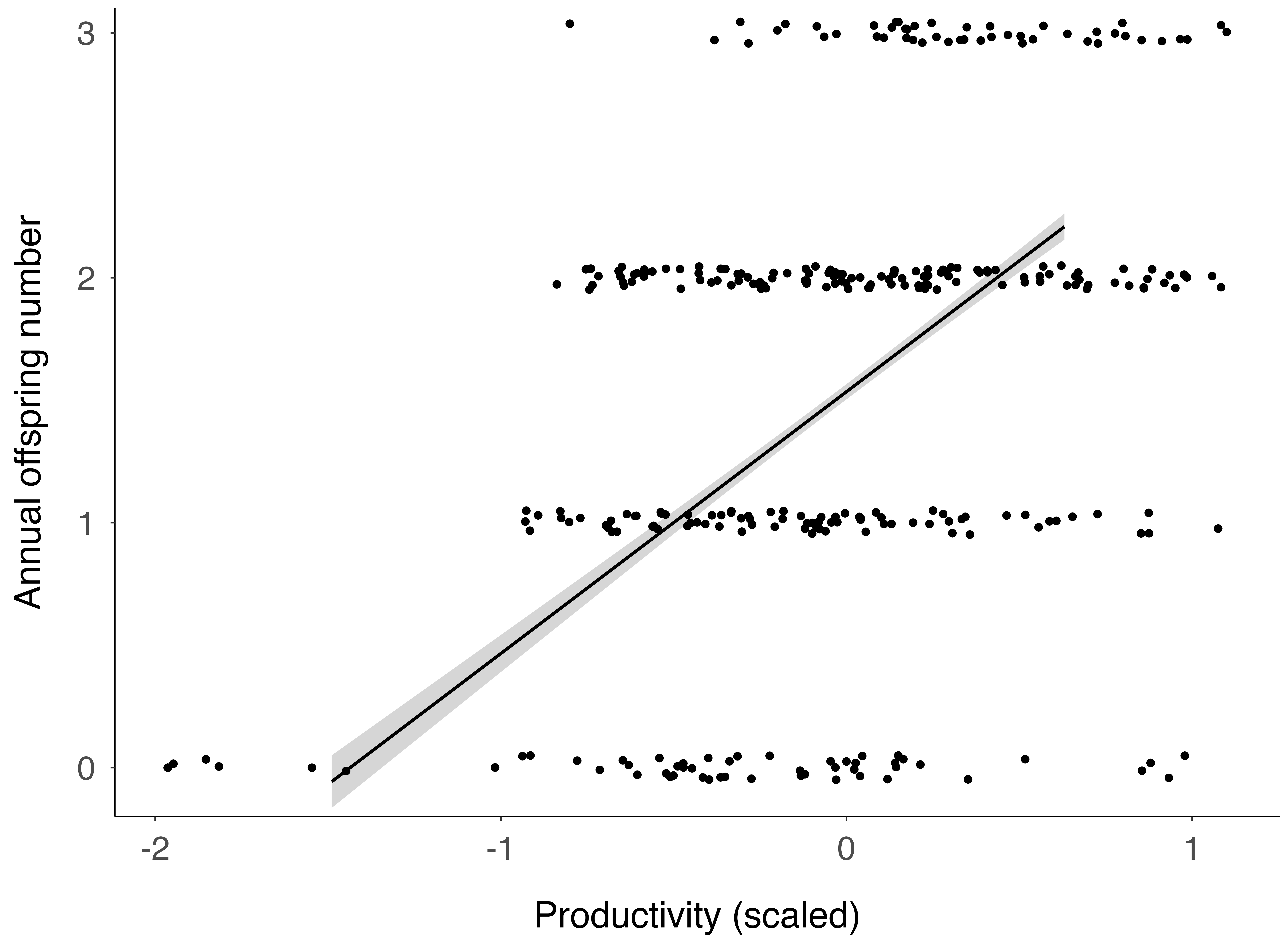
**

**Figure S3:** Annual offspring number (number of fledglings per breeding attempt) in relation to δ age (within-individual age effects, in years), and in interaction with the early-life environment (mean annual wind intensity of the year of birth) in male Eleonora’s falcons. Data were categorized into three early-life (EL) environmental quality groups using the 25th percentile, the median, and the 75th percentile. Age effects were binned and averaged for visualisation purposes, note that this does not reflect each with-individual’s senescence pattern but mean patterns. Only the smoothed regression line for the group with a trend is plotted (high-quality early-life environment x δ^2^ age: β = -0.32, SE = 0.18, Z = -1.82, P – value = 0.0698). Lines and shaded areas represent the smoothed predicted values estimated from the model (both morphs pooled) with upper and lower 95% confidence intervals as the grey shaded areas. Key: -1SD = high-quality EL, mean = benign EL, +1SD = low-quality EL. Raw data points are shown.


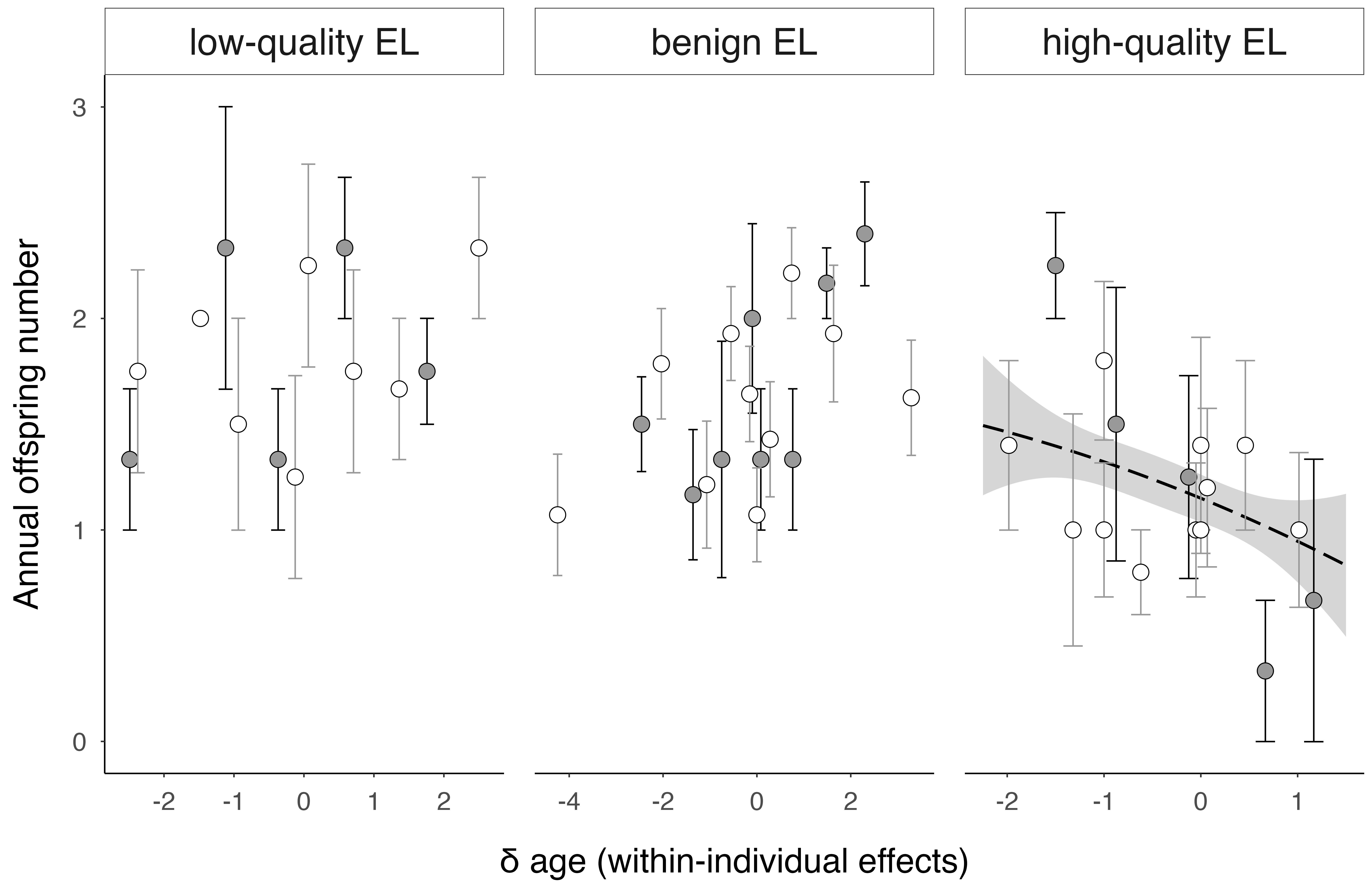


**Figure S4:** Annual offspring number (number of fledglings per breeding attempt) in relation to the adult-life environmental conditions (mean annual wind intensity of each breeding year) in interaction with the early-life environment (mean annual wind intensity of the year of birth) in male Eleonora’s falcons. Data were categorized into three early-life environmental quality groups using the 25th percentile, the median, and the 75th percentile. Lines and shaded areas represent the smoothed predicted values estimated from the model (both morphs pooled) with upper and lower 95% confidence intervals as the grey shaded areas. Key: -1SD = high-quality early-life environment, mean = benign early-life environment, +1SD = low-quality early-life environment. Raw data points are shown, colour coded by morph (grey= dark, white =pale).


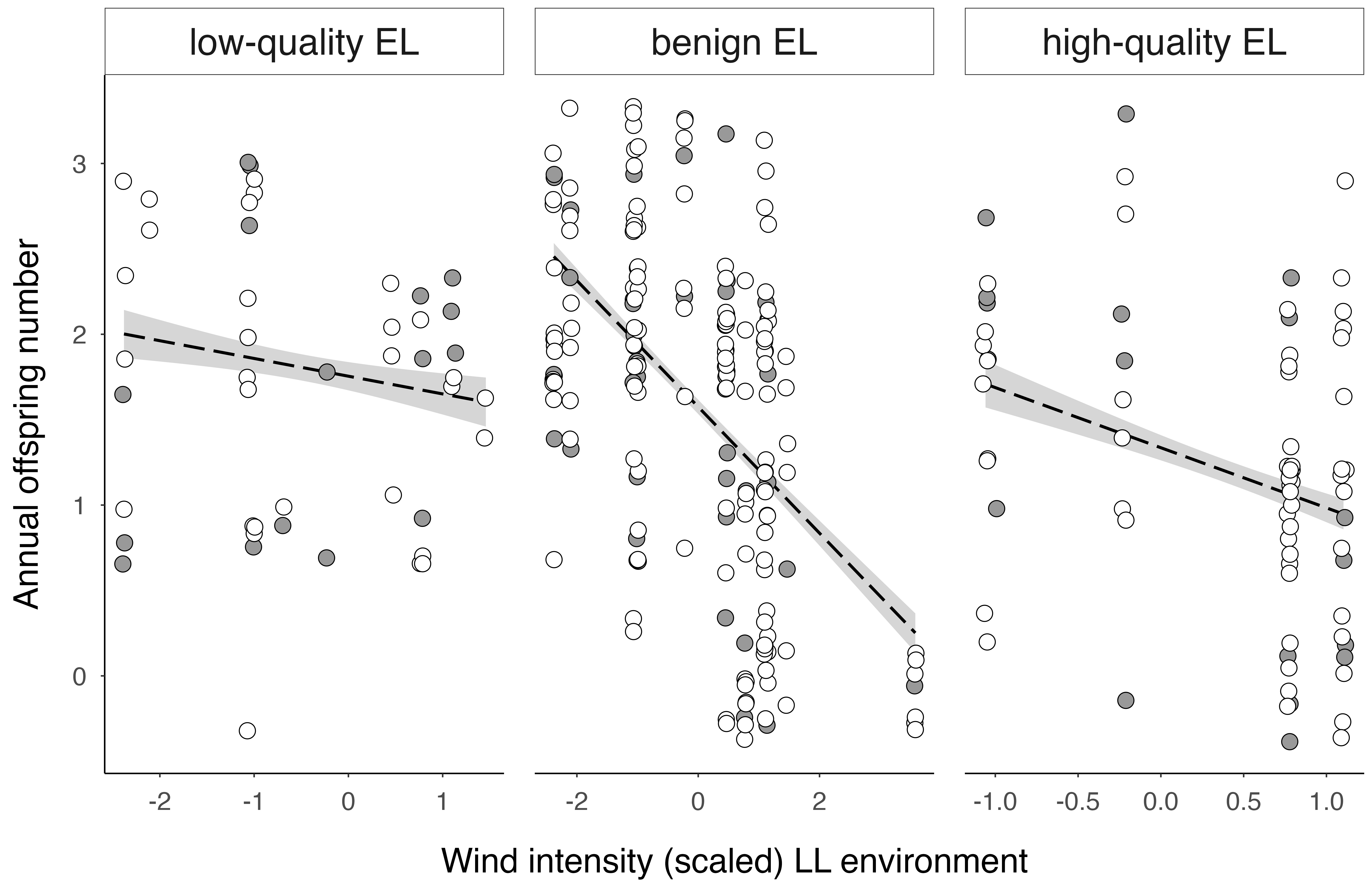

Supplement: arag024_Supplementary_Data [file arag024_supplementary_data.docx]
